# Overview of Promising Solutions in Subsurface Sounding Equipment

**DOI:** 10.3390/s23208461

**Published:** 2023-10-14

**Authors:** Ivan V. Bryakin, Igor V. Bochkarev, Vadim R. Khramshin, Vadim R. Gasiyarov

**Affiliations:** 1 Laboratory of Information and Measuring Systems, National Academy of Sciences of the Kyrgyz Republic, Bishkek 720010, Kyrgyzstan; bivas2006@yandex.ru; 2 Department of Electromechanics, Kyrgyz State Technical University named after I. Razzakov, Bishkek 720010, Kyrgyzstan; elmech@mail.ru; 3 Power Engineering and Automated Systems Institute, Nosov Magnitogorsk State Technical University, 455000 Magnitogorsk, Russia; hvrmgn@gmail.com; 4 Department of Automation and Control, Moscow Polytechnic University, 107023 Moscow, Russia

**Keywords:** metal detector, hidden subsurface object, inductive and penetrating radar techniques, combined subsurface sounding techniques

## Abstract

This overview analyzes current advances in the equipment for detecting various subsurface metal and metal-containing objects. Various metal detector types are discussed alongside their operation principles, properties, and capabilities. Following the analysis of conventional metal detectors, promising design and technical solutions are explored, implementing new physical metal detector operation principles that have not been used before for this equipment class. The information provided allows for evaluating new metal detector concepts developed to improve the sensitivity and accuracy of detecting equipment.

## 1. Introduction

In the course of operation, repair, construction, and rescue, some specific problems arise associated with the quick detection and precise positioning of various metal Subsurface Objects (SOs). Such objects can be exemplified by underground utilities components: water and gas pipelines [1], heating networks [2], electrical cables, and various underground cable structures [3,4]. SOs may also include hidden metal and metal-containing objects, e.g., reinforcement inside walls, buried scrap metal, treasures, hidden shells or mines, etc. Thus, SOs may be relatively small in size in terms of the depth of their underground location, causing additional technical challenges in their detection [5,6] and identification [7,8]. Detecting metal objects in food products can also be attributed to such problems [9,10].

Obviously, fulfilling tasks assigned to the services concerned without an accurate detection of SOs in the work area becomes fundamentally more complicated and sometimes even impossible. It should be noted that as a rule, the location of hidden utilities is fixed in relevant documents. However, underground utilities are often moved, e.g., during maintenance, or their location changes due to various natural phenomena; thus, even official documents are not always reliable. Moreover, maps commonly reflect the general SO location, while the work requires more detailed SO positioning data, especially in urban areas. Other SOs are either located irregularly (scrap metal) or hidden deliberately (e.g., mines). In this regard, existing detection and identification equipment is being continuously improved, and new, more accurate and sensitive equipment is being developed. Generalizing the results and techniques of these studies may form a basis and guideline for further research of metal detectors used in subsurface sounding.

This research is aimed at highlighting the current advances in the metal detectors’ development for subsurface sounding and identifying possible ways to improve them qualitatively. Note that any SO detection system comprises two units:

1. Primary measuring transducer—a sensor (metal detector) designed to detect SOs, e.g., by generating a scanning primary field, recording a secondary SO-reradiated field and converting it into an electrical output data signal;
2. Secondary measuring transducer—an electronic unit for receiving and processing data signals and transmitting data via an external interface.

Obviously, the detection system sensitivity and accuracy are determined by the capabilities and parameters of both these units. However, the existing variety of such units and the ways of their design and technical improvement cannot be considered in full within a single limited study. Thus, the authors only focused directly on analyzing the induction-type metal detector design.

The paper structure is as follows: Section 2 provides a general classification of modern metal detectors and briefly discusses their operation principles and the specifics of various techniques. Section 3 analyzes the induction balance technique specifics and considers the antenna module design options. Section 4 analyzes approaches to the problem of classifying detected metals. Section 5 considers the impact of the host medium on the metal detector operation. Finally, Section 6 analyzes promising metal detectors built on the basis of new physical operation principles.

It should be emphasized that this review is not aimed at a detailed numerical comparison of the technical indicators of various well-known metal detectors; it analyzes the principles of metal detector structure as well as possible ways and means of improving their metrological properties through the use of fundamentally new approaches towards the structure and implementation of their primary measuring transducers.

## 2. Classifying Modern Metal Detectors

To detect and fix underground utilities and metal objects in the upper layers of the earth’s crust (host medium), various methods are applied. They are based on recording the detected object response to the impact of various artificial physical fields, e.g., electromagnetic, acoustic, seismic, thermal, ionizing, etc. [11]. Under the impact of these fields, the SO response changes depending on both the exciting field type and the individual detected object parameters. 

**Figure 1:**
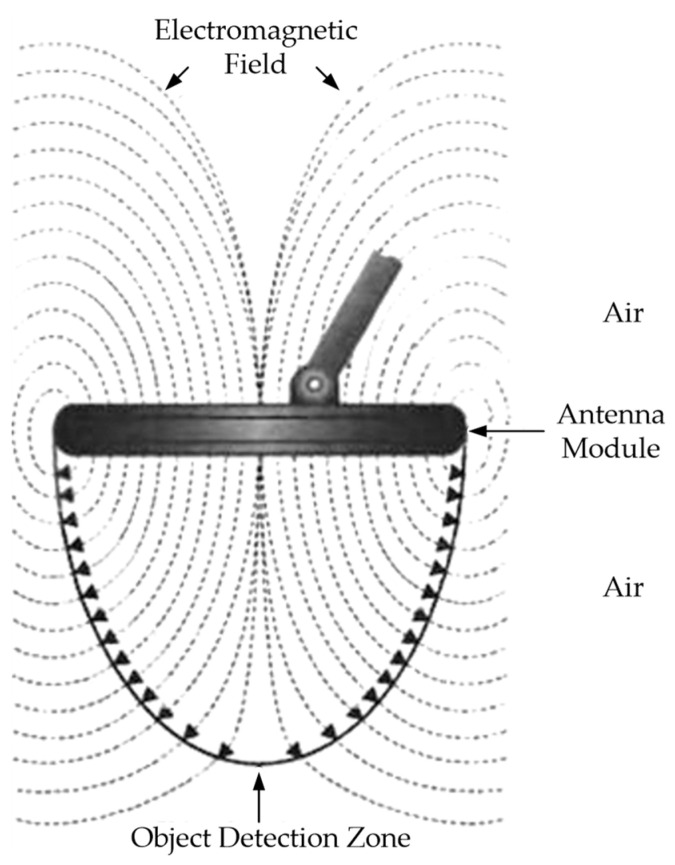
Electromagnetic field of the search coil.

**Figure 2:**
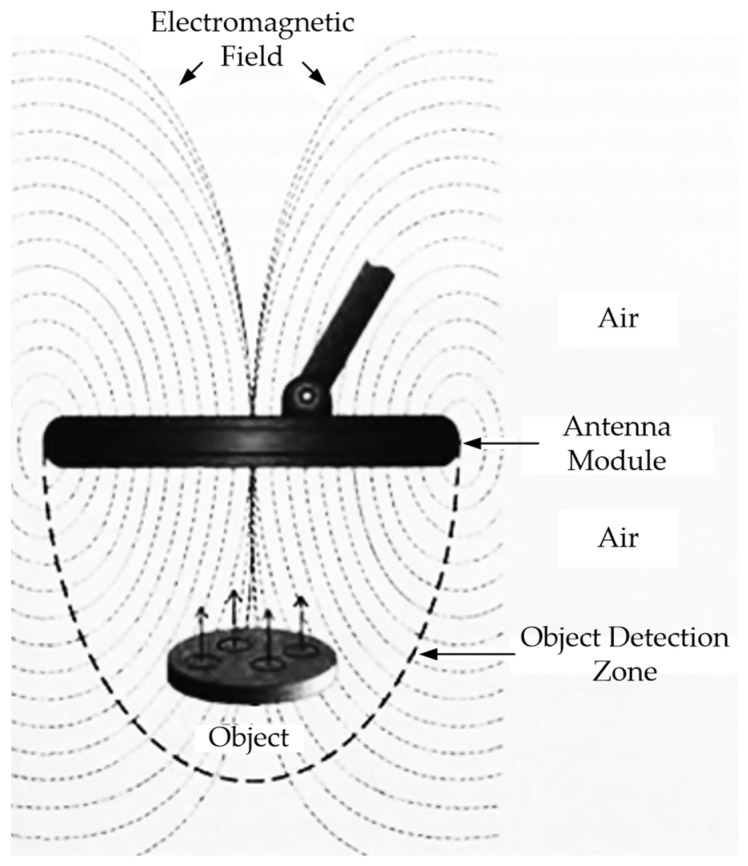
Reradiated electromagnetic field of a metal object.

The operation of the vast majority of metal detectors, consisting of an antenna module and an electronic information signal processing module, is based on electromagnetic phenomena. Their operation principle is that the alternating electric current flowing in the search coil generates an electromagnetic field around it that passes into the environment (Figure 1). If a metal object gets in the way of this field, so-called eddy currents occur on its surface, which form their own electromagnetic field (reradiated) weakening the field of the search coil (Figure 2).

The electronic module of the metal detector, using a search coil, detects this weakening of the field caused by the presence of a metal object under the search coil; after appropriate processing of the recorded signal, it reports the presence of this metal object.

Although the types of metal detectors are different, the principle of their operation is the same. Since the invention of these devices (modern development of metal detectors began in the 1920s), much of their basic operation has not changed, but technology advances have led to the development of different metal detectors.

Modern metal detectors mainly use the following techniques: (1) inductive technique (either a passive low-frequency one consisting in detecting an alternating magnetic field generated by a current-carrying conductor or an active high-frequency technique consisting in detecting a magnetic field electromagnetically induced in the utility); (2) a penetration radar technique based on emitting electromagnetic pulses and recording signals reflected from various objects. The widespread use of Metal SO Detectors (MDs) is explained by their certain advantages: high sensitivity and mobility, simplicity and ease of use, low weight and small dimensions, etc. Their efficiency largely depends on the Antenna Module (AM) design, which is, in fact, an inductive converter of the electromagnetic field into an electrical signal [12,13,14,15].

Depending on the signal generated and processed in a particular device, metal detectors can be divided into two categories:

1. FD (Frequency Domain) comprises a group of metal detectors based on detection techniques using continuous sinusoidal signals in a resonant or frequency mode and implementing electromagnetic impedance measurements that allow for estimating the following: (a) the response of the antenna’s input impedance to the probed medium’s electromagnetic properties; (b) the response of the electromagnetic field parameters to the medium’s intrinsic or surface impedance when the field propagates in the soil or above ground.

Most of the well-known metal detectors belong to the FD category and use the principle of estimating the electromagnetic field changes under the metal object impact. A common feature of such devices is an active coil generating an electric field.

2. TD (Time Domain) comprises a group of metal detectors based on radiophysical detection techniques using a pulse signal with subsequent estimating changes in its parameters over time. Thereat, the recurrence rate of pulses generated in these devices ranges from several tens to hundreds of hertz.

**Figure 3:**
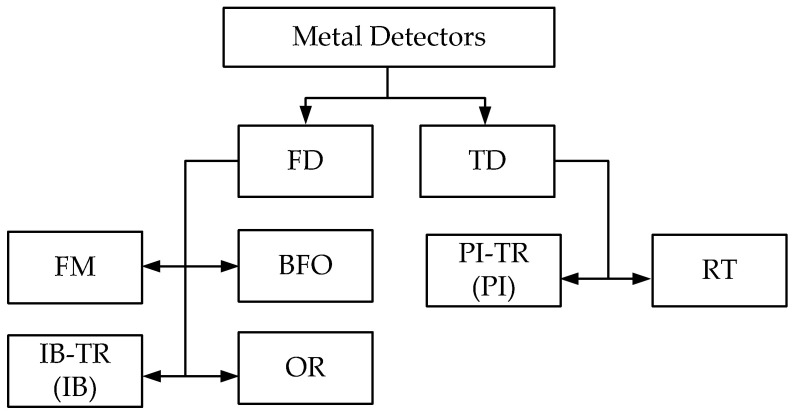
The Metal Detector Classification Option: FD—Frequency Domain; TD—Time Domain; FM—Frequency Meter; BFO—Beat Frequecy Oscillation; IB-TR—Induction Balance-Transmitter Receiver; OR—Off Resonance; PI-TR—Pulse Induction-Transmitter Receiver; RT—Radiolocation Technique.

Several fundamental criteria are generally used to classify modern metal detectors [16,17]. One of the most convenient ones is the criterion based on estimating sounding signals [18]. Figure 3 shows an option of classifying metal detectors based on this criterion.

***The BFO (Beat Frequency Oscillation)*** technique uses a single-loop antenna. It is based on the reradiated electromagnetic wave receiver’s input impedance response to the probed medium’s electromagnetic properties. It involves recording the (pulsation) frequency difference of signals from two generators, the frequency of one of which is unchanged, and the frequency circuit of the other contains a sensor in the form of a loop antenna.

**Figure 4:**
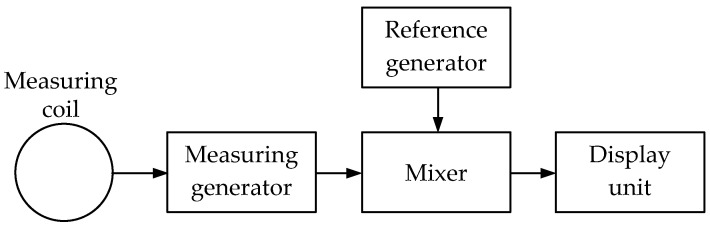
Simplified flowchart of a metal detector operating on the BFO principle.

Figure 4 shows a simplified flowchart of a metal detector operating on the BFO principle. Two same-frequency generators (measuring and reference) are used while the frequency of the reference one is unchanged. The measuring generator’s working circuit coil is simultaneously detecting (emitting) and measuring one. When a metal object occurs in the coverage area of the detecting coil electromagnetic field, the measuring generator frequency changes. The changed frequency signal is fed to the mixer, where it is mixed with the reference frequency signal. As a result, a beat frequency signal is generated at the mixer output, which is fed to the display unit.

Beating metal detectors generally use acoustic, pointed, and LED indicators.

It should be noted that in BFO metal detectors, the beat frequency falls within a low-frequency range, closer to the lower limit of the human ear sound perception. This allows for significantly simplifying the display unit design since the beat frequencies of the main (measuring, detecting) and auxiliary (reference) generators can be analyzed by ear. However, the sensitivity of metal detectors based on the beat principle leaves much to be desired. However, their specifications would completely satisfy unpretentious users.

***The FM (Frequency Meter)*** technique relies on the principle of measuring the frequency deviation of the reference generator under the impact of metal objects within the detecting coil coverage area.

**Figure 5:**
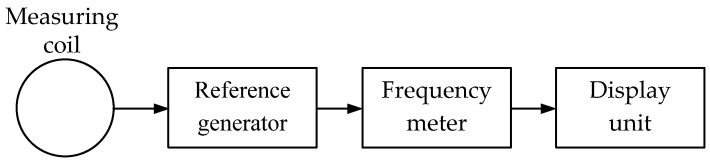
Simplified flowchart of a metal detector operating on the frequency meter principle.

Figure 5 shows a simplified flowchart of a metal detector operating on the frequency meter principle.

When a metallic object occurs in the coverage area of the measuring coil electromagnetic field, the reference generator circuit resonant frequency changes. A frequency meter based on a microcontroller evaluates this change. The frequency deviation magnitude and sign depend not only on the object size and location depth but also the metal type. The processed data are fed to the display unit.

Note that FM metal detectors are more sensitive than, e.g., BFO ones.

***The OR (Off Resonance)*** technique uses a single-loop antenna. It is based on the emitted electromagnetic wave receiver’s input impedance response to the probed medium electromagnetic properties. The technique is based on the principle of estimating the change in the circuit coil (loop antenna) signal amplitude, the resonant frequency of which is close to that of the reference generator signal fed.

**Figure 6:**
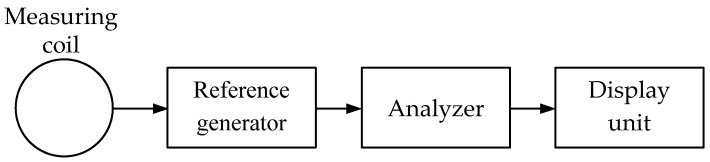
Simplified flowchart of a metal detector operating on the off resonance principle.

Figure 6 shows a simplified flowchart of a metal detector operating on the off-resonance principle.

The measuring coil is an integral part of the oscillatory circuit, the resonant frequency of which differs slightly from that of the reference generator. When a metal object occurs in the coverage area of the measuring coil electromagnetic field, the resonant frequency of this circuit changes. Depending on the metal in the device (ferrous or non-ferrous) coverage area, the circuit frequency either increases or decreases. Thereat, the oscillation amplitude of the reference generator changes accordingly, which is evaluated by the analyzer. As a result, the analyzer generates a control signal for the display unit.

***The IB (Induction Balance)*** technique implements the transmitter-receiver principle, which involves recording the signal, reradiated by a metal object under an alternating magnetic field of a transmitting loop antenna, by a receiving loop antenna. It is based on the response of the electromagnetic field parameters to the medium’s intrinsic or surface impedance when an electromagnetic wave propagates in the soil or above ground.

The design and relative position of the primary and receiving antennas depends on the operating frequency ranges:

(a) VLF band (3 kHz ÷ 30 kHz)—IB/TR/VLF (Induction Balance/Transmitter-Receiver/Very Low Frequency); at least two combined wide-span 2D loop antennas are used, one of which is transmitting and the other is receiving;
(b) LF band (30 kHz ÷ 300 kHz)—IB/TR/LF (Induction Balance/Transmitter-Receiver/Low Frequency); it uses at least two concentric (combined) coplanar loop antennas, one of which is transmitting and the other is receiving;
(c) MF band (300 kHz ÷ 3 MHz)—IB/TR/MF (Induction Balance/Transmitter-Receiver/Medium Frequency); it uses at least two combined loop antennas with perpendicular axes, one of which is transmitting and the other is receiving;
(d) HF band (3 MHz ÷ 30 MHz)—IB/TR/HF (Induction Balance/Transmitter-Receiver/High Frequency); it uses at least two spaced loop antennas with perpendicular axes, one of which is transmitting and the other is receiving.

In TR-IB-type devices, in the course of detection, the receiving coil receives a signal initiated by eddy currents arising in a metal object under the impact of a continuous transmitting signal. The analysis of the received signal parameters (e.g., amplitude and phase shift) is the source of information on the presence and features of metal objects in the device coverage area.

**Figure 7:**
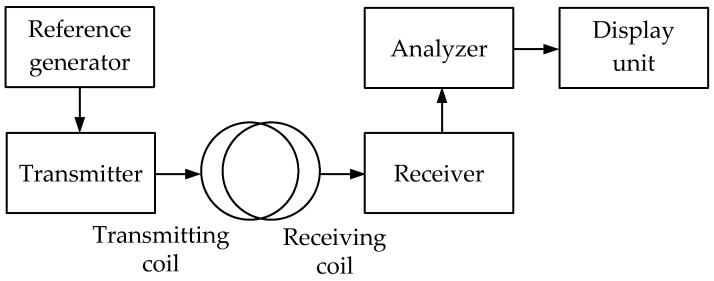
Simplified flowchart of a metal detector operating on the transmitter-receiver principle.

Figure 7 shows a simplified flowchart of a metal detector operating on the transmitter-receiver principle.

The transmitting signal generated by the reference generator is fed to the transmitter and further to the transmitting coil (loop antenna). When a metal object occurs in the transmitting coil coverage area, eddy or surface currents are initiated on its surface under the impact of, e.g., a VLF signal. These currents induce the secondary signal, received by the metal detector’s receiving coil. After the receiver, this signal is fed to the analyzer evaluating its parameters. Based on the analysis, an appropriate data signal is generated for the display unit.

**Figure 8:**
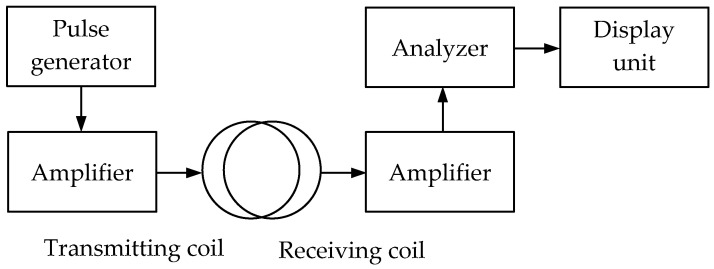
Simplified flowchart of a pulse metal detector with 2D antennas.

***The PI (Pulse Induction)*** technique uses a single-loop antenna with alternating transmission and reception modes or two combined wide-span 2D loop antennas. It is based on the response of the electromagnetic field parameters to the medium’s intrinsic or surface impedance when an electromagnetic wave propagates in the soil or above ground. The reradiated signal is analyzed, generated in a metal object by eddy surface currents under the impact of an external electromagnetic field. Figure 8 shows a simplified flowchart of a pulse metal detector implementing the pulse induction technique.

The pulse signal generated by the pulse generator is amplified and fed to a transmitting coil initiating an alternating electromagnetic field. When a metal object occurs in this field coverage area, eddy currents arise from time to time on its surface under the pulse signal impact. These currents induce the secondary signal, which is received by the receiving coil, amplified, and fed to the analyzer. It should be noted that due to the self-induction, the secondary signal will be longer than the pulse emitted by the transmitting coil. Thereat, the secondary pulse signal trailing edge parameters are used for analysis, followed by forming data for the display unit.

When a special decoupler or switch is available, the pulsed metal detector uses a single coil instead of transmitting and receiving ones to alternately transmit and receive signals.

**Figure 9:**
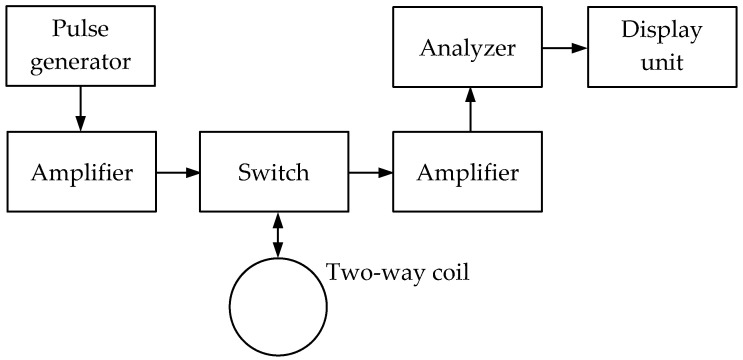
Simplified flowchart of a pulse metal detector with a single antenna.

Figure 9 shows a simplified flowchart of a pulse metal detector with a single coil.

The pulse signal generated by the pulse generator is amplified and fed to the loop antenna initiating a pulsing electromagnetic field in the surrounding space through the switch alternating the antenna’s transmission and reception operating modes. When a metal object occurs in this field coverage area, eddy currents frequently arise on its surface under the impact of a pulsing field, which is a source of a secondary (reradiated) electromagnetic field. This secondary electromagnetic field is received by the receiving coil (loop antenna) while generating a corresponding electrical signal in it, which is amplified and fed to the analyzer. The signal processing data from the analyzer output is fed to the display unit, where it is visualized.

It should be pointed out that the key advantages of pulse metal detectors are relatively high sensitivity and the simple coil design. However, the circuit solutions of individual units (such as a pulse generator, a switch, or an analyzer) remain quite complex. Such devices also use microprocessors with appropriate software.

***The RT (Radiolocation Technique***) technique is based on evaluating the parameters of a microwave signal reflected from a metal object. In this case, the reflected signal amplitude depends not only on the object size but also the material conductivity. Along with the amplitude, the reflected signal delay is also analyzed, which indicates the metal object’s depth.

Depending on the penetrating radar signal structure, two PR techniques are distinguished: continuous emission of oscillations and the pulse method.

In the object radar detection, the continuous microwave signal emission is based on the Doppler effect, where the frequency of the signal arriving at the receiving device varies depending on the relative transmission speed between the transmitter and receiver. Thus, it is virtually not used in subsurface sounding.

A variation of the method of continuous radiation of oscillations is the SFCW (Stepped Frequency Continuous Wave radar) method, which uses a step law of modulation of the frequency of the sounding signal [19]. A radar implementing this method operates at several sequentially varying frequencies. At each of these individual frequencies, the phase of the echo is measured. This type of radar is intended mainly for interferometric measurements, but it can also be used in certain cases in the metal detector mode. However, its relatively complex circuit design and advanced software, and thus its relatively high cost, make its use impractical in the mode of a conventional metal detector.

The pulsed radiation technique is commonly used, where short-term powerful ultrahigh-frequency (UHF) oscillation pulses are periodically emitted with a duration of τ ≈ 0.1 ÷ 1 μs, and in the so-called rest interval between them (generally *T* ≈ 1 ms) pulse echo signals reflected from objects are received. It is generally assumed here that the sounding pulse and the reflected echo signal durations are equal.

Currently, in the RT technique, two types of pulses are used as sounding signals: 1—a high-frequency radio pulse; 2—pulse without carrier, i.e., monopulse (video pulse) consisting of one or more current oscillations in the antenna with a relative spectral band close to 1. The pulse radar design depends on whether the transmitter and receiver are located in the same (combined, monostatic radar) or different (multi-position, bistatic radar) places.

Along with the compact design, the combined pulse radar’s advantage is that synchronizers important for the pulse radar can be concentrated in a central synchronization unit. Thus, the internal delays at the switches can be short. It should be noted that due to the antenna switch, such a radar’s antenna can be used for both emitting and receiving signals.

**Figure 10:**
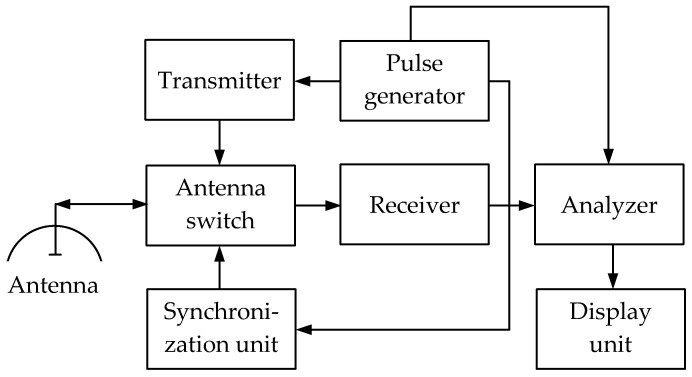
Simplified flowchart of a monostatic PR metal detector.

Figure 10 shows a simplified flowchart of a monostatic PR metal detector using radio pulses.

The pulse signal from the pulse generator modulates the transmitter signal emitted by the antenna. Reaching the object, the transmitted signal is reflected from it. The reflected signal is received by the antenna and then fed to the analyzer through the antenna switch and the receiver. Along with this, the pulse generator signal is also fed to the analyzer.

The analyzer compares both signals and evaluates the difference while forming data for the display unit. Thereat, the detected object depth data are formed after evaluating the reflected signal delay, and the object size data are formed by the signal amplitude.

**Figure 11:**
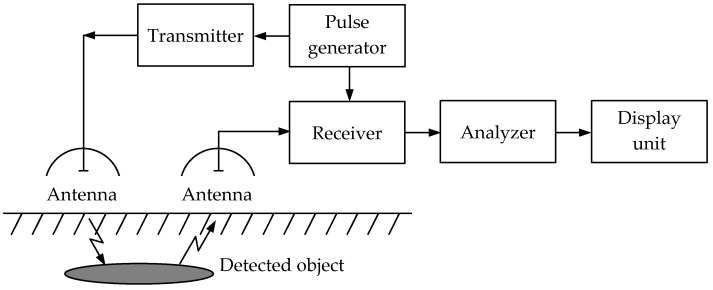
Simplified flowchart of a bistatic PR metal detector.

In a bistatic pulse radar, the receiver has its own antenna located at a distance from the transmitter. This determines the advantage of no need for complex measures to protect the receiver from high transmitter power. Figure 11 shows a simplified flowchart of a bistatic PR metal detector using radio pulses. Having two antennas allows for greater isolation between the receiving and transmitting channels and eliminating switches. Thereat, the pulse is emitted by the transmitting antenna, propagates in the soil, is reflected from objects located mainly under the transmitting antenna, and further received by the receiving antenna. The receiving antenna is located next to the transmitting antenna, and they generally form a unified antenna module.

Travassos X. L. et al. [20] describe the fundamental theory of improving antennas for GPR systems and their optimization. The authors considered the antenna as a two-port transducer system characterized by a transmission function that may be frequency-dependent and have a non-linear phase response. It is shown that for the best resolution, the antenna should have a wide frequency band. The maximum detection depth decreases as the electromagnetic wave frequency increases. In reality, most subsurface radar systems operate at frequencies below 5 GHz. The antenna should also have good and stable performance over the entire operating range, including directional pattern, gain, impedance matching, and low dispersion. High gain and small beam width are fundamental to target detection in close proximity.

The pulse GPR antenna calculation issues are considered in [21], performing a comparative analysis of GPR antennas, proposing a new type of antennas for powerful pulse geoprobes, and assuming the nature of dispersion distortions of reflected signals.

Reference [22] provides an overview of the detection and mapping of underground utilities using GPR. The overview covers GPR data collection and survey procedures, preprocessing, and characterizing using image processing and machine learning techniques.

Bilik Y., Haridim M., and Bilik D. propose a way to improve the lateral resolution of detecting underground rectilinear objects using GPR with two transmitting antennas fed in an antiphase [23]. It is shown that the proposed technique allows for significantly improving both the relative area of reflectivity (RAR) of rectilinear objects and the lateral resolution when detecting narrow underground objects. This technique allows for achieving a high horizontal resolution of about 0.2 m at depths of 20–30 m. RAR dependencies on the object depth and width have been calculated and provided. This technique is applicable for both orthogonal and any other scan pattern.

Reference [24] considers three GPR application areas, which are promising and will be further developed: GPR beam tomography, integration of GPR with other geophysical techniques, and the use of GPR for solving geocryological problems. The examples of implementing various techniques for collecting and processing data to solve geological and technical problems are given.

## 3. Specifics of the Induction Balance Technique

There are common requirements for metal detectors: manufacturability, reliable operation, resistance to small changes in the geometric measurement conditions, etc. All known versions of metal detectors have certain drawbacks that significantly reduce their detection capabilities and do not meet the set requirements to some extent.

The most significant drawback is the excitation of a direct signal by the primary magnetic field in the receiver, which changes in the course of detection, leading to systematic noise and measurement errors. The direct signal magnitude and change limits depend on both the chosen geometry of the transmitter-receiver system (combined or spaced transmitting and receiving antennas) and that geometry violation degree.

The induction balance technique is known as the most widely used one. To improve the induction balance equipment sensitivity in detecting subsurface objects, the primary field signal induced in the receiving antenna should be suppressed or significantly reduced. To do this, geometric compensation of the primary field is provided by the mutual orientation or spacing of the transmitting and receiving antennas; electrical compensation of the primary field signal induced in the receiver is applied by adding complex electronic compensators to the measuring equipment. All these measures significantly complicate the equipment and methodology of the work, thereby increasing its cost.

The limitations defining the reasonable application area for each of the specified metal detector modifications are associated with both the metal detector features and the sounding problems being solved (detection, detailed localization of objects in depth), as well as the object features (urban underground utilities, intercity cable lines, pipelines).

It should be noted that, as a rule, metal detectors use either a rigid compact arrangement of the transmitting and receiving antennas, which allows for the geometric compensation of the primary field, or sufficiently spaced transmitting and receiving antennas, the rigid mutual orientation of which is not required while the primary field impact is reduced due to increased installation base and differential connection of two receiving antennas. As a rule, loop transmitting and receiving antennas are used.

The main difference between metal detectors is the size of the base between the transmitting and receiving antennas, which is considered small if it is comparable with the loop antenna dimensions, and large if it significantly (tens and hundreds of times) exceeds them.

**Figure 12:**
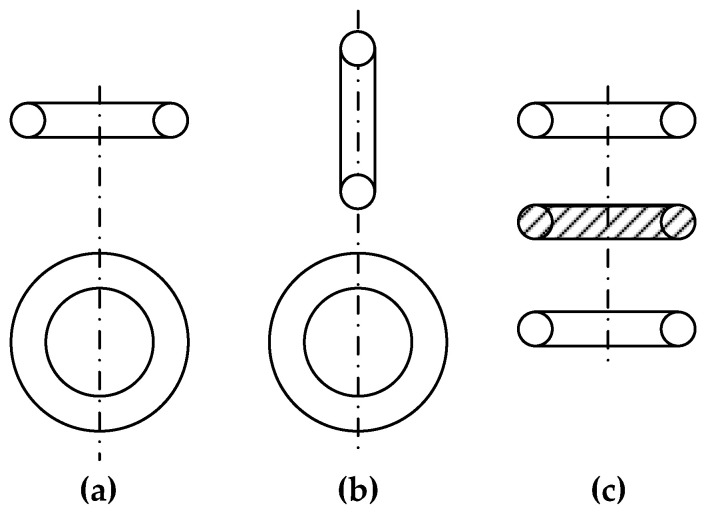
The AM coil mutual arrangement versions: (**a**)—coils with perpendicular axes; (**b**)—coils with crossing axes; (**c**)—a system of one transmitting (in the center) and two opposite receiving coils.

In urban environments with specific problems of detecting local SOs, the size and location depth of which are commensurate with the metal detector loop antenna dimensions, it is advisable to use quicker metal detectors with a small base for preliminary detection. Herewith, a necessary condition for the effective solution of these problems is, primarily, providing a reliable and time-stable primary field compensation at the receiving antenna location or the signals excited in the receivers by this field. Two basic mutual arrangements of coils with perpendicular and crossing axes (Figure 12a,b) are most commonly used to solve these problems, where the signal is not directly transmitted from one coil (transmitting) to another (receiving).

There are also metal detectors containing several appropriately electrically connected coils (more than two), e.g., Figure 12c shows a system of one transmitting (in the center) and two opposite receiving coils regarding the signal induced by the transmitting coil. Ideally, the receiving coil system output signal is equal to zero since the EMFs induced in the coils are mutually compensated.

Metal detectors with coplanar coils (i.e., located in the same plane) are of particular interest. This is explained by the general use of such metal detectors to detect subsurface physical objects, and the metal detector’s antenna module (AM) can only be brought closer to the ground surface if its coils are coplanar. Moreover, such antenna modules are generally compact and fit well into protective disc-shaped housings.

**Figure 13:**
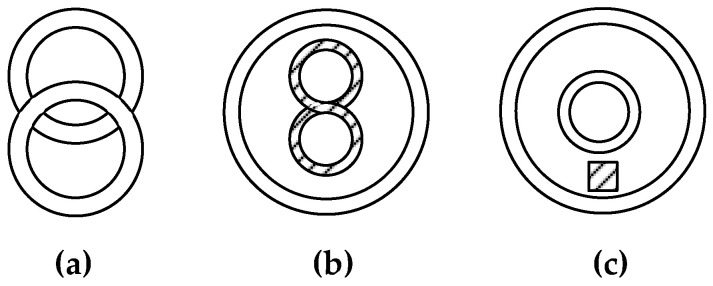
Coplanar versions of the AM coil mutual arrangement: (**a**)—basic versions coils; (**b**)—8-shaped coils; (**c**)—coplanar axially symmetric coils.

Figure 13a,b shows the basic versions for the mutual arrangement of coplanar AM coils. The diagram in Figure 13a shows the mutual arrangement of the coils where the total magnetic flux through the surface, bounded by the receiving coil, is equal to zero. In the diagram in Figure 13b, one coil (receiving) is 8-shaped, so the total EMF, induced on the receiving coil’s half-turns located in one wing of “Figure 8”, compensates for a similar total EMF induced in another one.

Various designs of AM metal detectors with coplanar axially symmetric coils are also known (Figure 13c). In this case, the receiving coil is inside the transmitting coil. EMF induced in the receiving coil is compensated for by a special transformer drawing off part of the transmitting coil signal.

**Figure 14:**
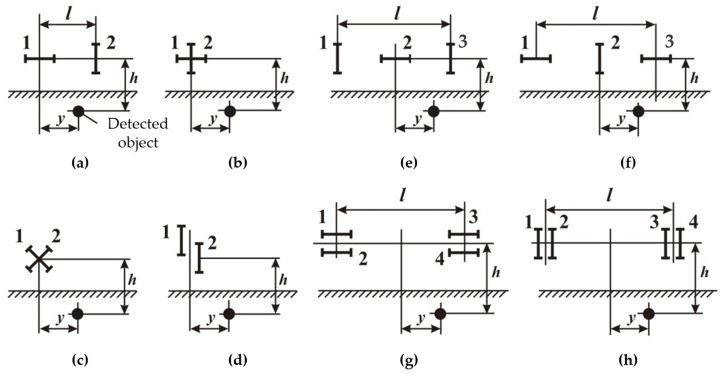
Antenna module versions: (**a**–**c**)—various non-differential AMs with mutually orthogonal loops; (**d**)—non-differential AMs with mutually coplanar loops; (**e**,**f**)—various three-frame differential AMs; (**g**,**h**)—various four-frame differential AMs.

Figure 14 schematically shows various versions of AM metal detectors with a small base, where the loops are indicated by straight line segments perpendicular to the reception directions, exciters have even numbers, and receiving antennas have odd numbers. For easy analysis and comparability of the results, the design parameters are assumed to be the same for the loops in all AMs. AMs are shown conventionally in the profile plane perpendicular to the object.

The small base AMs differ by the loop number (two, three, four, etc.), connection (opposite, aiding), and relative position (orthogonal, coplanar). Each of these features affects both the secondary and primary induced signals and, therefore, cannot serve as the sole or preferred classification criterion.

There are non-differential (Figure 14a–d) and differential (Figure 14e–h) AMs. The latter ones stipulate for the opposite connection of identical loops 1, 3 (with the aiding connection of identical excitatory loops 2, 4). Differential AMs comprise three-frame (Figure 14e,f), four-frame (Figure 14g,h), and non-differential ones—those with mutually orthogonal (Figure 14a–c) and coplanar (Figure 14d) loops. Within these subgroups, differences in the loop location relative to the object and the spacing in the profile plane can be considered. Loop spacing perpendicular to the profile plane, i.e., along the object, has virtually no effect on the secondary induced signal and thus is not considered.

The key differences in the AM (Figure 14a–h) sensitivity, resolution, and accuracy of object localization are defined by a comparative analysis of the profile characteristics *f*(ξ), where ξ = *y*/*h* —a dimensionless coordinate along the profile perpendicular to the object longitudinal axis, where *y*—distance between the object vertical axes and the AM frame of the metal detector (Figure 12a) and *h*—distance between the object horizontal axes and antenna module frames (Figure 12a) (object depth of occurrence).

As an example, we give the formula for calculating the profile characteristics for the AM option shown in Figure 12a:

$$f_{a}(\xi )=\frac{\xi }{\left(1+\xi^{2})[1+{\left(\xi +k\right)}^{2}\right]},$$

where *k* = *l*/*h*, *l*—basic distance between the AM frames (Figure 14a). 

By way of example, at *l* = 0.75 m and *h* = 1.5 m, antenna modules of the ‘d’, ‘c’, and ‘a’ types (Figure 14) [*f_d_*(ξ)]_max_ = 1, [*f_c_*(ξ)]_max_ = 0.5, and [*f_a_*(ξ)]_max_ = 0.45, respectively, have the best sensitivity. The antenna modules of the ‘b’ and ‘f’ types [*f_b_*(ξ)]_max_ = 0.33 and [*f_f_*(ξ)]_max_ = 0.23, respectively, are noticeably inferior to them in sensitivity. Modules of the ‘c’, ‘g’, and ‘h’ types with [*f*(ξ)]_max_ < 0.1 have the worst sensitivity.

With respect to the detected object (cable, pipeline), the profile characteristics of the ‘a’ type of AMs are odd and asymmetric. The object’s route is determined by the lateral extrema obtained during the forward and reverse travel along the profile as the middle of the distance between them. The profile characteristics of the ‘b’, ‘g’, and ‘h’ types of AMs are odd but symmetrical. In this case, travelling along the profile in one direction is enough to determine the route by lateral extrema (maximum and minimum). The profile characteristics of the ‘c’-‘f’ types of AMs are symmetrical and even, where *f_c_*(ξ), *f_d_*(ξ) and *f_f_*(ξ) give the maximum and *f_e_*(ξ) gives the minimum readings over the object route. Due to this, the ‘c’-‘f’ types of AMs are distinguished by high resolution and provide convenient and quick tracing with a satisfactory error (about ±0.2 ÷ 0.3 m) when localizing objects in plan. 

AMs of the ‘b’ and ‘e’ types give the smallest error in determining the object depth by the extreme profile characteristic points (about ±0.35 m at a depth of 1.5 m), other AMs give large errors, and the ‘d’ type is generally unusable for determining depths.

Differential AMs of the ‘f’-‘h’ types are distinguished by better noise immunity; thereat, their structure is more complex, and configuring is more labor-intense (symmetrization is required).

Based on the combination of the basic technical and operational characteristics (sensitivity, noise immunity, simplicity and ease of tracing, object localization accuracy), non-differential AMs of the ‘c’ and ‘b’ types and differential AMs of the ‘f’ type can be recommended for use. Herewith, since AMs of the ‘c’ and ‘b’ types differ only in their orientation regarding the object (rotation angle), they can be considered as two positions of the same AM, one of which (‘c’) is preferable in terms of sensitivity and the ease of tracing, and another (‘b’)—the accuracy of determining the object depth.

In general, regarding AMs for the FD metal detectors, we can state the following:

1. The AM coil system with perpendicular axes is preferable for detecting relatively small physical objects than that with crossed axes. Ceteris paribus, the first system, has a slightly higher sensitivity. It also allows for defining the exact object detection direction much more easily.
2. The considered AM coil systems have an important property that allows for estimating the distance to small objects by nulling the reflected signal at a distance to the object equal to half the base.
3. Ceteris paribus (the coil dimensions and number of turns, the reception path sensitivity, the magnitude and frequency of current in the emitting coil), the sensitivity of the metal detector operating on the ‘transmission-reception’ principle virtually does not depend on its base, i.e., the distance between the coils.

In [25], Tumanski S. gives an overview of various sensors with different types of induction coils. The author compared and generalized the methods of designing air and ferromagnetic-core coils, analyzed the frequency properties of sensor coils, and described various techniques for processing output signals. Special types of inductive sensors are described, such as the Rogowski coil and gradiometer, vibration coil, tangential field, and needle sensors. The sensors are shown to only be sensitive to the flow perpendicular to their main axis; therefore, three mutually perpendicular coils should be used to determine all directional components of the magnetic field vector. It is also noted that air-core coil designs are widely used in NDT as eddy current sensors, e.g., to detect defects, and are often made as a flat planar coil in the form of a printed board or according to thin film technology.

In [26], Tang Z. and Carter L. considered the impact of coil positioning errors on metal detector sensitivity. It has been shown that errors in positioning the coil inside the head may degrade the detector’s performance.

In [27], Takahashi K. provided the data on the differences between the single coil, double D-coil, and other metal detector types in the field. A linear correlation between the SO location accuracy and the sensitivity profile has been found for single-coil detectors. The author shows that a detector with a smaller search head gives more accurate results than those with large ones, which makes them generally preferable for SO detection. However, scanning the specified area takes more time. Oval coils and a double-D configuration are well-suited to solve this problem. The author has shown that the sensitivity profile is elliptical in vertical section, and its width narrows with distance from the coil; therefore, when raising the search head above the ground, a smaller part of the sensitivity zone can be used to determine the location. Reference [28] also reviews the efficiency and accuracy of detecting subsurface objects in the field, considering the search head size.

Kyoo Nam Choi proposed a two-channel metal detector with two sets of perpendicularly oriented sensor antennas [29]. The author proved that a single-channel metal detection sensor does not provide the required sensitivity within a wide range of metal sizes from millimeters to centimeters. Thus, cascading sensors with different resolutions are required. However, the second sensor cannot be located next to the first one since its electromagnetic wave will strongly affect the first sensor. The proposed two-channel metal detector is formed by cascading two sensors with different resolutions, having perpendicular antennas to minimize physical noise, and the optimal way of detecting a signal while suppressing the sensor interference is studied.

## 4. Identifying Subsurface Objects

Most metal detectors fix only the metal object presence without differentiating its type. Graziella Bedenik et al. [30] proposed an approach to detecting and classifying metals. The technique is based on the change in the coil’s inductive resistance when it approaches a metal object. This effect is used to detect and classify ferrous and non-ferrous objects based on phase measurements. The results obtained show that this approach allows for creating a simple, cheap and affordable electronic design of a metal detector capable of detecting and classifying ferrous and non-ferrous metals.

Caorsi S. et al. [31] proposed a technique for identifying subsurface objects based on the differential evolutionary approach. The method implements the inverse scattering problem. Based on Green’s function for a half-space, integral equations have been obtained, relating the object’s dielectric properties to patterns of a scattered electric field.

Yamazaki S., Nakane H., and Tanaka A. developed the SRPM technique for the simultaneous evaluation of the electrical and magnetic properties of a spherical object by vector measurement of the impedance difference of two round solenoid coils, one with and another without the sample [10,32]. The impact of metal object properties such as size, conductivity, and permeability on the amplitude and phase of the output signal of a metal detector with a transmitting coil in the center and two coaxially arranged receiving coils on the sides has been theoretically analyzed. Based on this technique, using a spherical sample to simplify the analysis, an equation was derived for estimating the vector voltage induced by a metal object in the receiving coil.

In [33], Nelson C.V. considered the basic technologies for detecting metal objects using electromagnetic induction techniques. It is stated that since each conductive object has a unique time decay characteristic, a library of conductive object signatures can be developed. When a hidden metal object is detected, its time signature can be compared to those in the library, and if a match is found, the object can potentially be classified. Classification allows for distinguishing between potential threats and non-hazardous objects.

In [34], Lu Yilin and Wei Dong studied a metal detector based on finite element analysis to obtain the relationship between the output current amplitude and the metal object material, size, and location.

Mlambo P. et al. [35] proposed a technique based on inverse electromagnetic induction problems to define the depth, shape, size, and electrical properties of the material (conductivity and permeability) of a hidden object with finite dimensions. Two common techniques proposed to estimate target parameters by measurements are model fitting and pattern recognition. Model fitting involves developing a mathematical model to describe the secondary reradiated fields as a function of the source parameters with subsequent maximum likelihood estimation (such as a least-square fit) to determine the parameter values that best fit the measurements. A numerical model in the MLE procedure can also be used instead of an analytical equation while increasing computational complexity. Model fit is obviously limited by the availability of an applicable model, and most geometries do not have simple models. Recognizing SOs involves comparing some electromagnetic parameters of an unknown object with those of a known one to find out whether they match or not. It also involves the analysis of response curves in the complex plane and their simplification by extracting the appropriate set of basic and additional features. The increasing number of objects requires large libraries. Thus, the problems become less solvable as the libraries get bigger.

However, as the authors have shown, despite their success, these approaches cause problems since they are based on a database previously created for recognition, which in fact limits recognition to the data volume in this database. Building existing systems and reusing existing applications is also difficult.

## 5. The Host Medium Impact

Obviously, detecting subsurface objects is significantly affected by the properties of the host medium in which the detected object is located. Thus, theoretical and experimental studies are required to find out which electromagnetic properties are important and to what extent they affect the performance of various metal detectors. A lot of studies have been devoted to this issue.

Researcher Yogadhish Das et al. thoroughly studied the impact of the host medium properties on the metal detector operation based on available geophysical and non-destructive testing data. In [36], the soil magnetic susceptibility was simulated as complex and frequency-dependent. Simplified analysis versions were applied for three selected cases of practical importance, i.e., non-conductive soil with constant susceptibility, non-conductive soil with frequency-dependent susceptibility, and non-magnetic soil with constant conductivity. The analysis of these cases has shown that the metal detector performance is affected more by the medium’s magnetic properties than electrical conductivity.

In [37], Das Y. considered the impact of the soil’s electromagnetic properties on metal detectors. That paper provides an analysis based on available geophysical and non-destructive testing data. The host medium is simulated as a half-space with a real and frequency-independent electrical conductivity but a frequency-dependent complex magnetic susceptibility. The author has shown that the soil magnetic properties affect the continuous wave and pulse-induction detectors differently. In some cases, representing practical scenarios for detecting a subsurface object, the signal from the soil may dominate that from the object, making it difficult to detect. It has also been shown that magnetic soil may change the spectral response of the target. Thus, it is concluded that, contrary to existing practice, object detection techniques should consider the electromagnetic parameters of the host medium.

In [38], Druyts P., Craeye C. and Acheroy M. describe a general model for calculating the EMI sensor’s response to magnetic soil in both the time and frequency domains. The model can be applied for arbitrary inhomogeneities and topography of the soil, as well as arbitrary shapes, orientations, and positions of sensor coils. The model is built based on the concept of a head sensitivity map, which can be used to characterize the sensor head depending on the sensor coil shape, size, and position. A general model has been developed for the case when the soil’s magnetic susceptibility is sufficiently low. A simpler model valid for only homogeneous soils has also been developed. Both models show good agreement with each other and available analytical solutions.

Authors in [39] also consider the impact of magnetic soils on the EMI sensors. Double D-heads have been shown to provide good soil compensation, however, assuming approximate homogeneity over a larger volume of soil. To quantify the impact, the concept of the Volume of Influence (VoI) has been introduced for the EMI sensors. This contributes to a better understanding of the magnetic soil response to the EMI sensor and the soil heterogeneity impact on soil compensation. The VoI is first defined as the volume producing a fraction of the total response of a homogeneous half-space. Since this basic definition is not appropriate for sensor heads with intrinsic soil compensation, a generalized definition is proposed. These definitions still do not yield a unique VoI, and a constraint should be introduced to reach uniqueness. Two constraints are investigated: one yielding the smallest VoI and another the layer of influence. Those two specific volumes of influence have a number of practical applications that are discussed. The smallest VoI is illustrated for typical head geometries, and we prove that, apart from differential heads such as the quad head, the shape of the smallest VoI is independent of the head geometry and can be computed from a far-field approximation. Moreover, quantitative head characteristics are provided and show, among others, that double-D heads allow for good soil compensation, assuming, however, approximate homogeneity over a larger volume of soil. The effect of soil inhomogeneity is further discussed, and a worst-case VoI is defined for inhomogeneous soils.

## 6. Promising Types of the FD Metal Detectors Category

### 6.1. Ferrite Antenna-Based AM Metal Detector 

**Figure 15:**
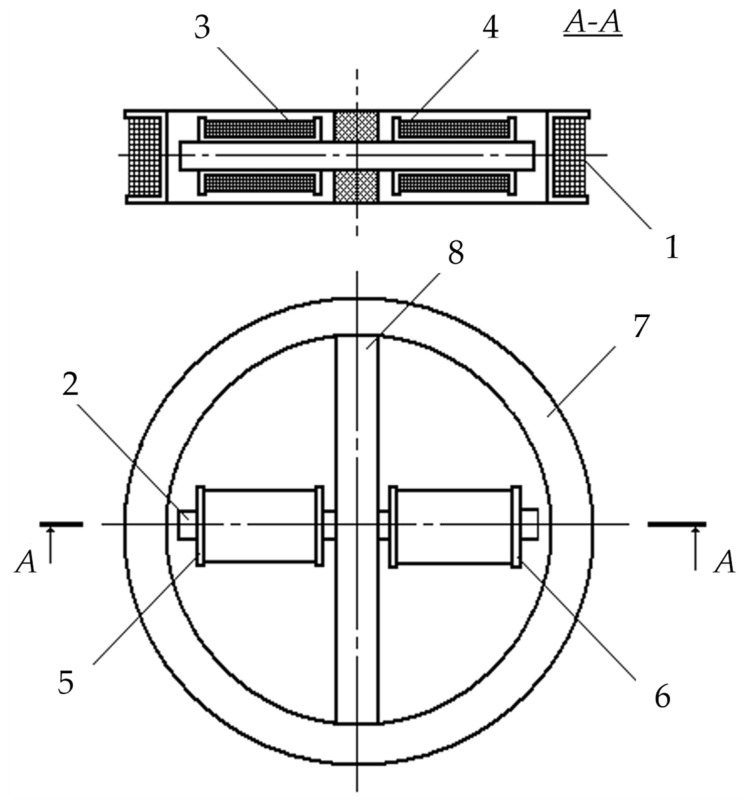
The AM metal detector design.

In the existing FD metal detectors, the coils of the AM receiving circuits do not have ferromagnetic cores capable of increasing the magnetic flux [15]. A ferromagnetic core in the AM receiving antenna will allow for reducing the number of turns and improving sensitivity and the signal-to-noise ratio. In this regard, the possibility of using a Ferrite Antenna (FA) as an AM receiving antenna is of particular interest. Figure 15 shows a general version of the design of such a ferrite antenna-based AM [40].

AM consists of a radiating Loop Antenna (LA) 1 placed on a ring frame 7 and a ferrite antenna composed of three elements: a ferrite cylindrical core 2 and receiving coils 3 and 4. Inside frame 7, in its plane, a base dielectric prism 8 is diametrically located, the length and height of which are equal to the frame’s inner diameter and thickness, respectively. Prism 8 has a symmetrically located transverse through-hole in the center, in which core 2 is fixed. Receiving coils 3 and 4 are located at opposite ends of core 2 symmetrically to the main symmetry axes of prism 8. Such a design solution stipulates for a mutual orthogonal arrangement of transmitting coil 1 and receiving coils 3, 4. With such a spatial orientation of the transmitting and receiving coils, the condition of their mutual inductance coefficient initially equal to zero will be met. The possibility of axial travel of receiving coils 3 and 4 along core 2 allows for providing the required initial compensation for the primary field signal. The mutual arrangement of receiving coils 3 and 4 on core 2 is set in two steps: first, roughly, by fixing each receiving coil in a certain place on core 2, and further, smoothly, using a special adjusting micro-screw.

**Figure 16:**
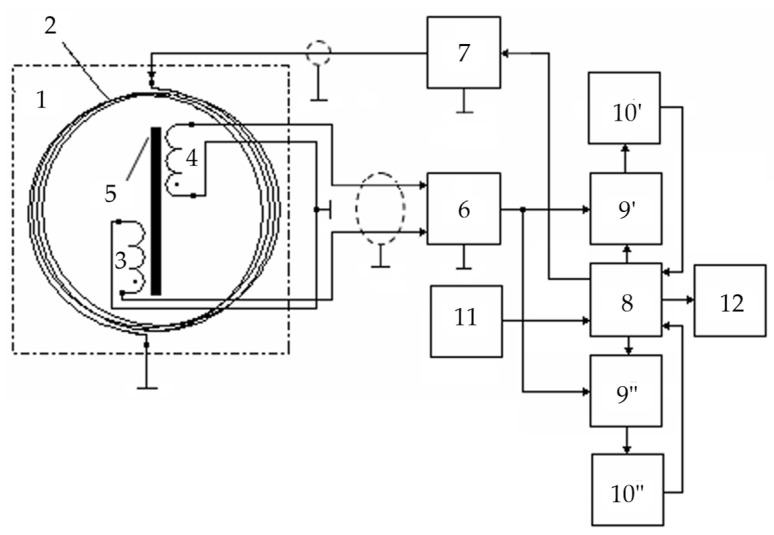
Flowchart of an FA-based metal detector.

Figure 16 shows a flowchart of a metal detector for the SO localization, where 1—combined AM consisting of radiating LA 2 and receiving coils 3 and 4, forming a FA together with ferrite core 5; 6—instrumental amplifier-adder; 7—audio frequency sinusoidal electrical signal generator; 8—computing unit; 9′—synchronous detector forming an in-phase measuring channel jointly with ADC 10′; 9″—synchronous detector forming a quadrature measuring channel jointly with ADC 10″; 11—data input device; 12—indicator.

The mutual spatial arrangement of the radiating loop antenna 2 and receiving coils 3 and 4 provides geometric primary field compensation (the mutual inductance coefficient of the antenna and receiving coils is zero), and the differential connection of coils 3 and 4 ensures mutual compensation for the EMF induced by external electromagnetic disturbances and additive noise. In this case, the total EMF at the output of amplifier 6 is equal to zero. This determines the metal detector’s invariancy to the impact of a uniform external background electromagnetic field and various destabilizing factors (changes in ambient temperature, humidity, etc.), i.e., increased noise immunity.

In the presence of a semiconducting host medium, the field of radiating loop antenna 2 magnetizes this medium causing a secondary polarized electromagnetic field (reradiated by the medium), already perceived by coils 3 and 4. Moreover, if the host medium is homogeneous, the compensation is not violated. The reflected electromagnetic wave vector is generally spatially oriented (polarized) differently from the sounding electromagnetic wave, leading to the emergence of corresponding horizontal components of this wave. At any time, signals are generated at the receiver output by electromagnetic waves reflected from the SO, spatially located in a certain way relative to the magnetic antenna’s receiving coils. In fact, electromagnetic waves reflected by the FA are recorded by its receiving coils 3 and 4 at, respectively, two spaced coordinate points within certain solid (viewing) angles ∆Ω′ and ∆Ω″.

When a foreign SO occurs in the host medium, the corresponding mutual induction effects emerge between it and each receiving coil. If the SO is spatially located asymmetrically relative to coils 3 and 4 (∆Ω′ ≠ ∆Ω″), the initial compensation is violated, and the corresponding voltage is generated at the output of instrumental amplifier-adder 6:

(1) $$U_{\Sigma }(t)=e^{\prime }(t)-e^{\prime \prime }(t)\neq 0.$$

The voltage at the output of instrumental amplifier 6 is the sum of two terms:

(2) $${\dot{U}}_{\Sigma }={\dot{U}}_{IP}+{\dot{U}}_{Q},$$

where ${\dot{U}}_{IP}=F(\mu_{SO})$ and ${\dot{U}}_{Q}=F(\sigma_{SO})$ are, respectively, the in-phase and quadrature components (with respect to the excitation voltage ${\dot{U}}_{0}$ LA); $\mu_{SO}$ and $\sigma_{SO}$ are, respectively, the SO magnetic permeability and specific electrical conductivity.

From amplifier 6, the signal is fed to the data inputs of synchronous detectors 9′ and 9″, the reference inputs of which are connected, respectively, to the outputs of the in-phase or quadrature reference voltages of computing unit 8.

The output signals of synchronous detectors 9′ and 9″ are described by the following Equation (3):

(3) $$\left\{\begin{array}{c} U_{SD}^{\prime }=r\times \left|{\dot{U}}_{\Sigma }\right|\times cos(\psi_{0}-\psi )\propto \mu_{SO}\;;\\ U_{SD}^{\prime \prime }=r\times \left|{\dot{U}}_{\Sigma }\right|\times sin(\psi_{0}-\psi )\propto \sigma_{SO}, \end{array}\right.$$

where ψ_0_ is the reference signal phase; ψ is the recorded data signal phase; *r* is the synchronous detector transmission coefficient.

Reference voltages for synchronous detectors are generated by unit 8 according to the set output signal parameters of generator 7, entered to unit 8 using a priori data input device 11, where the a priori data are the set parameters μ*_ef_*; μ∗; *w*; *S*; *U_m_*; ω; *H_m_*; *E_m_*; ψ_0_; *r*.

The outputs of synchronous detectors 9′ and 9″ are connected to the ADC inputs 10′ and 10″, respectively, which convert the analog input data signals into the corresponding digital signals of the in-phase and quadrature measuring channels, fed to the data inputs of computing unit 8.

According to the source data entered by data input device 11 and the recorded values of digital data signals of the in-phase and quadrature measuring channels, computing unit 8 performs algorithmic processing of these signals while detecting a subsurface object in the search area and identifying it by defining its magnetic permeability and electrical conductivity. A certain device calibration allows for defining the object’s depth. The computing unit 8 calculation results are displayed in the appropriate format on indicator 12.

The considered metal detector scans the studied host medium, fixing the degree of violation of the initial compensation of measuring coils, and the corresponding SO parameters are defined by the unbalance signal magnitude and sign. All the components required for the data signal processing algorithm are determined at the device’s preliminary calibration stage using the corresponding reference physical models of the host medium and SO.

The advantages of the proposed SO localization device are the effective implementation of the primary background electromagnetic field compensation conditions, the elimination of the impact of changes in the parameters of the ferromagnetic core and receiving coils on the receiving magnetic antenna characteristics, and significantly reduced inter-turn leakage in the receiving coils and the impact of external interference on them. The combination of the advantages of radiating loop antennas and the specifics of receiving magnetic antennas (high sensitivity), as well as their respective design, sets the proposed technical solution apart from its analogs and allows for significantly improving the sensitivity, selectivity, and manufacturability of the hidden object localization device [41]. Herewith, a ferromagnetic core in the AM receiving antenna allows for reducing the number of turns in it and improving its sensitivity and the signal-to-noise ratio.

### 6.2. Hybrid Subsurface Sounding Technique 

The hybrid subsurface sounding technique is of particular interest, which is implemented in an AM with a receiving single-section FA and LA combining the functions of the transmitting and receiving antennas [7,42]. An FA placed coplanarly inside a horizontal LA implements the technique of the response of the electromagnetic field parameters to the medium’s intrinsic or surface impedance, and the LA implements the technique of the receiving loop’s input impedance response to the probed medium’s electromagnetic properties. Herewith, the FA is structurally a single-section magnetic antenna placed coplanarly inside the horizontal LA.

**Figure 17:**
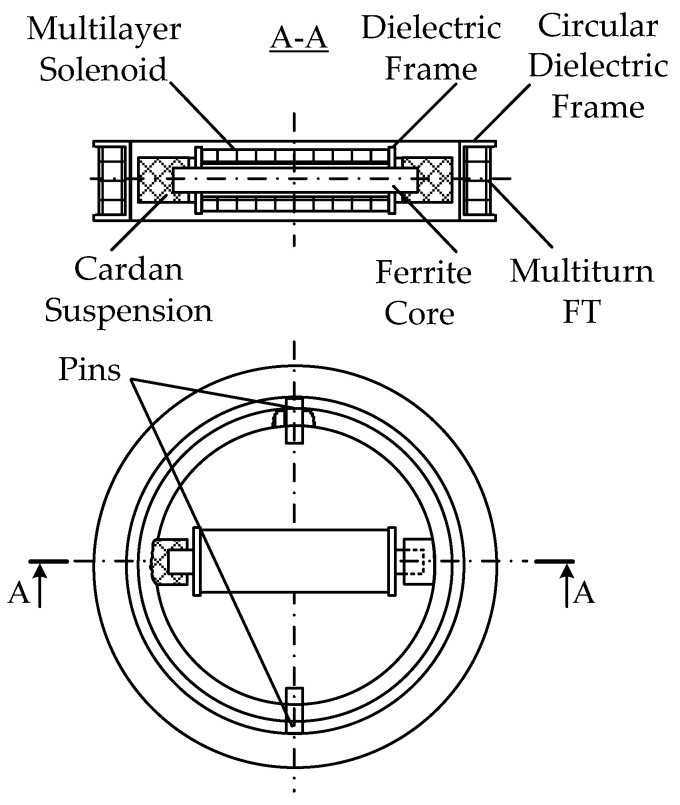
An AM based on a receiving FA and an LA with combined functionality.

Figure 17 shows a version of the AM design based on FA and LA with combined functionality. Such a structural solution stipulates for a mutually orthogonal arrangement of the LA and the FA’s receiving electric coil, which eliminates the direct connection between them and thereby geometrically compensates for the primary field. When implementing the hybrid subsurface sounding technique, data are recorded via three measuring channels:

- Two measuring channels of the receiving FA, which are used to measure, respectively, the current values of the voltage amplitude of the input signal’s active and reactive components;
- Measuring channel of transmitting-and-receiving LA, which is used to measure the current value of the LA exciting current amplitude.

Such data redundancy significantly improves the reliability and accuracy of the hidden metal object detection technique. This improves the efficiency of subsurface sounding in general.

**Figure 18:**
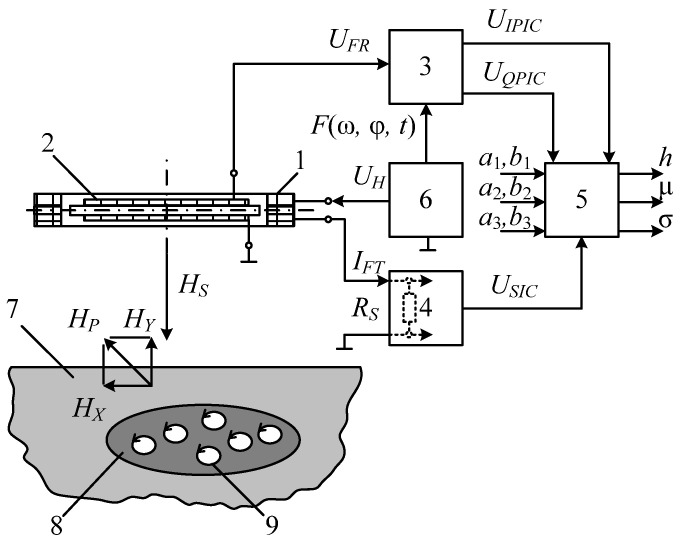
Flowchart of the hybrid subsurface sounding technique.

Figure 18 shows the structure flowchart of the hybrid subsurface sounding technique, where 1—transmitting-and-receiving LA; 2—receiving FA; 3—the procedure for recording and in-phase-quadrature converting FA signal (induction EMF) via the main measuring channel (MMC); 4—the procedure for recording and converting the reaction of the radiating LA impedance, caused by the SO electromagnetic properties, into an electrical signal via an additional measuring channel (AMC); 5—the procedure for algorithmic processing of measured data to define the SO parameters; 6—*U*_H_ harmonic signal generator exciting the primary electromagnetic field; 7—host medium; 8—SO; 9—eddy currents; *H_P_*—magnetic component of the primary electromagnetic field; *H_S_*—polarized magnetic component of the secondary electromagnetic field; *H_X_* and *H_Y_*—horizontal and vertical components of the polarized magnetic component of the secondary electromagnetic field; *R_SH_*—measuring current shunt; *a*_1_ and *b*_1_—the MMC static function coefficients for in-phase conversion; *a*_2_ and *b*_2_—the MMC static function coefficients for the quadrature conversion; *a*_3_ and *b*_3_—the AMC static function conversion coefficients; *U_FA_*—the FA data signal (induction EMF); *U_IPmmc_* and *U_Qmmc_*—in-phase and quadrature components of the *U_FA_* data signal, respectively; *U_AMC_*—the LA data signal; *F*(ω; φ; *t*)—synchronization of the MMC conversion with the time parameters of the harmonic signal exciting the primary electromagnetic field; σ and μ—respectively, the SO specific electrical conductivity and magnetic permeability; *h*—the SO depth [43,44].

When implementing the hybrid subsurface sounding technique, the operation frequency signal is fed from sinusoidal voltage generator 6 to transmitting-and-receiving LA 1, creating a primary electromagnetic field in the surrounding space. When SO 8 occurs in the host medium 7, it is magnetized by the primary field generating induced eddy current 9 in it, which generates a secondary (reradiated) electromagnetic field with a polarized magnetic component *H_S_*. The secondary electromagnetic field is recorded using spatially combined LA 1 and receiving FA 2.

The secondary field acts on receiving FA 2 and induces an induction EMF in it, generating a *U_FA_* signal. The *U_FA_* signal is converted into in-phase and quadrature electrical signals via the main measuring channel while the in-phase and reactive signals are proportional to, respectively, the specific electrical conductivity σ and magnetic susceptibility μ of SO 8. These in-phase and quadrature electrical signals are used as the MMC output signals. Thereat, the conversion via the main measuring channel is synchronized with the time parameters of the harmonic signal of sinusoidal voltage generator 6, exciting the primary electromagnetic field.

With a constant supply voltage of radiating LA 1, the electromagnetic field of eddy currents will increase its impedance and, as a result, decrease the strength of the current flowing in it. Thereat, the LA1 impedance will depend on the SO 8 eddy current magnitude and pattern of distribution in the host medium, i.e., the specific electrical conductivity σ and the SO 8 depth. In this case, the excitation current amplitude of loop antenna 1 is an informative parameter. The change in the impedance of radiating LA 1, caused by the electromagnetic properties of SO 8, is fixed via an additional measuring channel. To do this, an electrical signal proportional to the excitation current of radiating LA 1 is read by the AMC measuring current shunt *R*_SH_, further converted, and used as the AMC output signal. Then, the measured data are algorithmically processed to define the SO 8 parameters. To do this, the MMC and AMC output signals are jointly and algorithmically processed according to the source data set as the MMC static function coefficients *a*_1_, *b*_1_, *a*_2_ and *b*_2_, and the AMC static conversion function coefficients *a*_3_ and *b*_3_. According to the algorithmic processing results, the presence of subsurface SO 8 in the search area and its depth are recorded, and it is also identified by defining its magnetic permeability and electrical conductivity.

All the components required for the data signal processing algorithm are defined at the preliminary preparation stage by exposing the FA to a certain set of reference standards.

The SO 8 parameters are defined according to the following algorithm:

(4) $$\begin{array}{c} \sigma =\frac{b_{1}}{a_{2}b_{1}-a_{1}b_{2}}U_{{IP}_{MMC}}-\frac{b_{2}}{a_{2}b_{1}-a_{1}b_{2}}U_{Q_{MMC}};\\ \mu =\frac{a_{2}}{a_{2}b_{1}-a_{1}b_{2}}U_{Q_{MMC}}-\frac{a_{1}}{a_{2}b_{1}-a_{1}b_{2}}U_{{CIP}_{MMC}};\\ h=\frac{(a_{2}b_{1}-a_{1}b_{2})U_{AMC}-a_{3}b_{1}U_{{IP}_{MMC}}+a_{3}b_{2}U_{Q_{MMC}}}{b_{3}(a_{2}b_{1}-a_{1}b_{2})}. \end{array}$$

where *a*_1_, *b*_1_ and *a*_2_, *b*_2_ are, respectively, the MMC static quadrature and in-phase conversion function coefficients, defined at the stage of preliminary preparation of the host medium scanning by exposing FA 2 to a certain set of reference standards; *a*_3_, *b*_3_ are the LA 1 AMC static conversion function coefficients; σ and μ are the SO 8 electrical conductivity and magnetic permeability, respectively; *h* is the SO 8 depth in the host medium.

**Figure 19:**
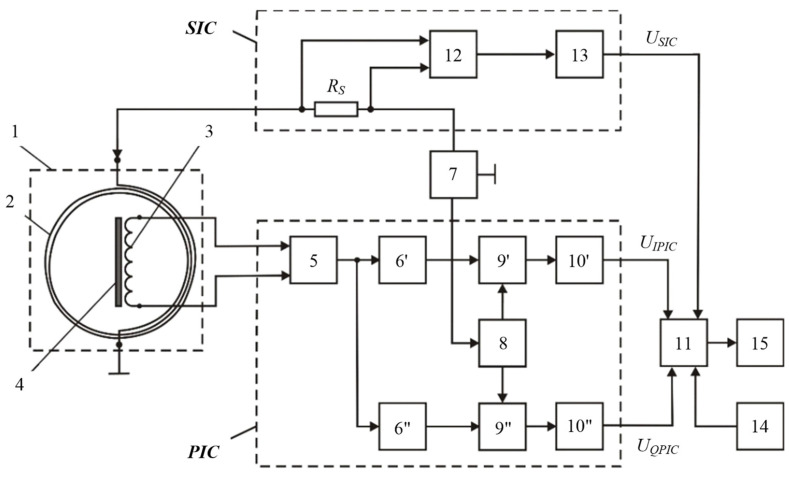
Structure flowchart of a metal detector with combined functionality.

Figure 19 shows a structure flowchart of a version of the hardware design of a metal detector implementing the combined subsurface sounding technique, where 1—AM; 2—LA; 3—receiving coil; 4—ferrite core; 5—measuring amplifier; 6′ and 6″—buffer amplifiers; 7—audio frequency sinusoidal voltage generator; 8—quadrature reference voltage shaper; 9′ and 9″—synchronous detectors (SD); 10′ and 10″—ADC units; 11—computing unit; 12—current meter; 13—ADC unit; 14—data input device; 15—indicator; *R_MSH_*—measuring current shunt. The AM elements 3 and 4 functionally form the FA. LA 2 is arranged orthogonally to the FA receiving coil 3, which provides geometric compensation for the primary field required for the FA [45].

This hybrid subsurface sounding technique and its technical implementation allow for defining the SO location and depth *h* in the host medium, as well as identifying the SO by σ and μ. parameters. All this significantly improves the informativity and efficiency of the proposed hybrid technique for detecting subsurface objects and expands the scope of its application.

**Figure 20:**
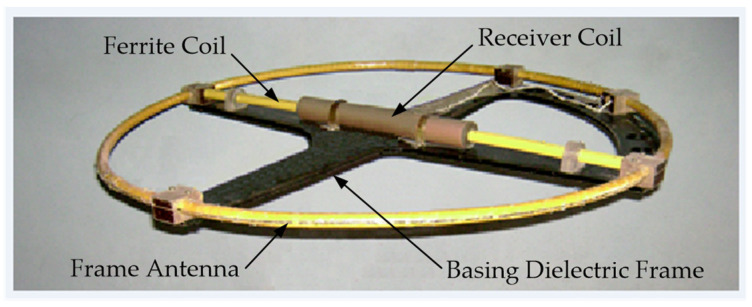
The AM frame with combined functionality.

A general view of the AM layout with combined functionality is shown in Figure 20.

**Figure 21:**
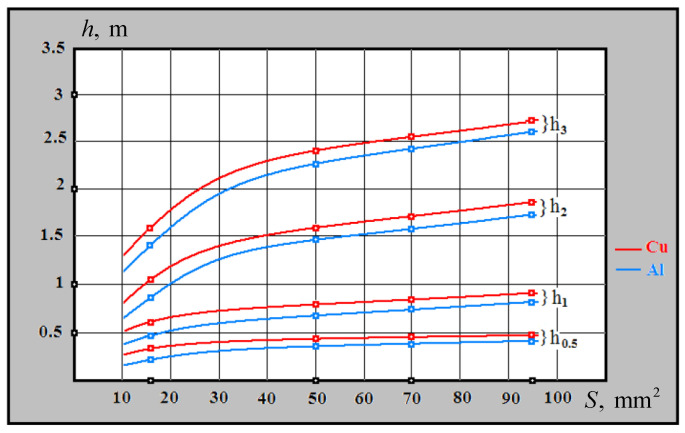
Measurements at different depths.

For example, Figure 21 shows the functionality of the experimental sample of this metal detector when detecting underground cables at the depth *h*_0.5_ = 0.5 m; *h*_1_ = 1 m; *h*_2_ = 2 m; h_3_ = 3 m (*S*—cross-section area of main cable cords (copper/aluminum). 

**Figure 22:**
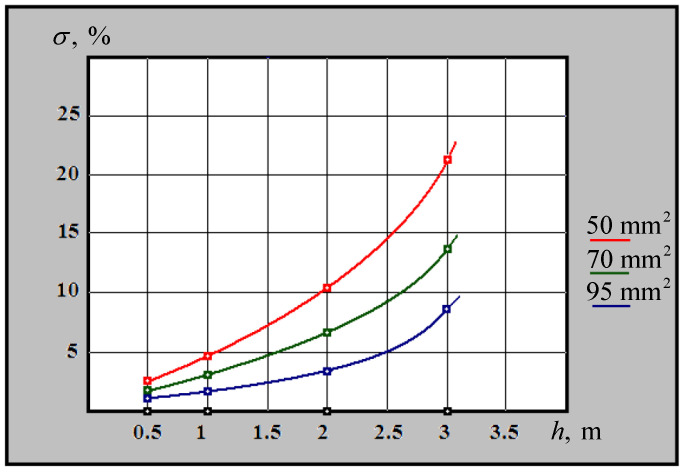
Distribution of relative depth detection error.

The distribution of the relative error in determining the depth of occurrence by a metal detector for cables with copper main conductors and different cross-sectional areas is shown in Figure 22.

### 6.3. Multiplied Subsurface Sounding Technique

The development of the hybrid subsurface sounding is the multiplied subsurface sounding technique, also implemented through the AM containing a receiving single-section FA and LA with combined functionality.

There are real limitations associated with the FA sensitivity threshold, which, in turn, leads to a sensitivity error, i.e., the FA sensitivity largely depends on its ferromagnetic core parameters (magnetic permeability) [8]. This factor significantly increases the likelihood of cases such as target drop-out or false responses.

**Figure 23:**
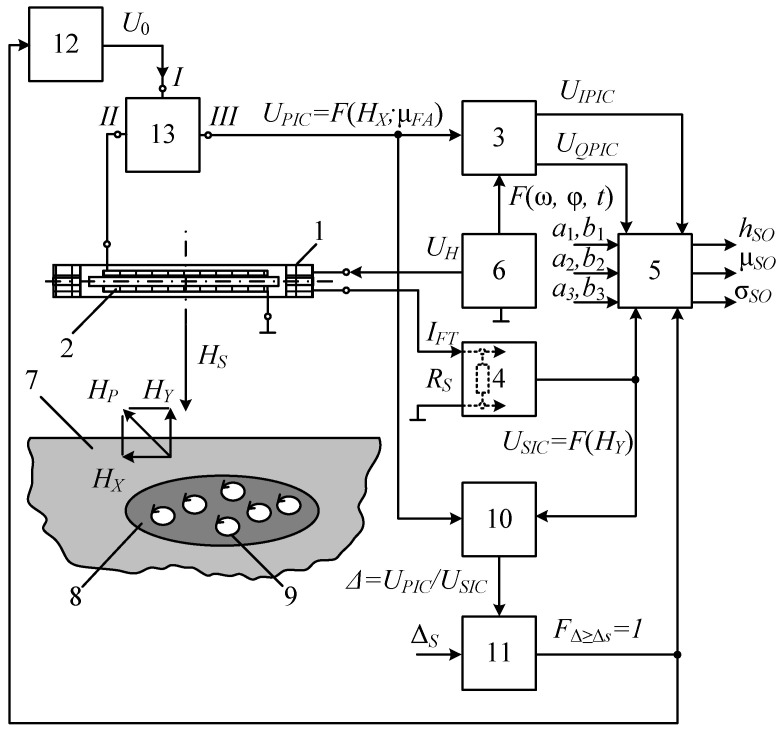
Structure flowchart of the multiplied detection technique.

Figure 23 shows the flowchart of the multiplied subsurface technique, where 1—transmitting-and-receiving LA; 2—receiving FA; 3—the procedure for recording and in-phase-quadrature converting the *U_MMC_* data signal via the MMC; 4—the procedure for recording and converting the reaction of the impedance of the radiating LA impedance, caused by the SO electromagnetic properties, into an *U_AMC_* electrical data signal via the AMC; 5—initiated procedures for recording data signals and algorithmic processing of measured data to define the SO parameters; 6—*U_H_* harmonic signal generator exciting the primary electromagnetic field; 7—host medium; 8—SO; 9—eddy currents; 10—the procedure for ratiometric conversion of two data signals *U_MMC_* and *U_AMC_*, where the conversion result is a quotient of the division Δ = *U_MMC_*/*U_AMC_*; 11—the procedure for comparing two values ∆ and ∆*_T_*, where ∆*_T_* is the threshold minimum to detect a foreign SO 8 in the host medium 7; 12—controlled source of the *U*_0_ constant scaled electric signal for biasing the FA core; 13—the procedure for Separation Filtering (SF) the *U_MMC_* data signal and the *U*_0_ constant scaled electric signal; μ*_FA_* is the magnetic permeability of the FA rod; *U_IPmmc_* and *U_Qmmc_*—in-phase and quadrature components of the *U_MMC_* data signal, respectively; *I_LA_*—the LA excitation current; *H_P_*—magnetic component of the primary electromagnetic field; *H_S_*—polarized magnetic component of the secondary electromagnetic field; *H_X_* and *H_Y_*—horizontal and vertical components of the polarized magnetic component of the secondary electromagnetic field; *R_SH_*—measuring current shunt; *a*_1_ and *b*_1_—the MMC static in-phase conversion function coefficients; *a*_2_ and *b*_2_—the MMC static quadrature conversion function coefficients; *a*_3_ and *b*_3_—the AMC static conversion function coefficients; $F_{\Delta \geq \Delta_{S}}(t)=1$—the initiation of the FA multiplied amplification mode and algorithmic definition of the SO parameters; *F*(ω; φ; *t*)—synchronization of the MMC conversion with the time parameters of the harmonic signal exciting the primary electromagnetic field; σ*_SO_* and μ*_SO_*—respectively, the SO specific electrical conductivity and magnetic permeability; *h_SO_*—the SO 8 depth [46].

**Figure 24:**
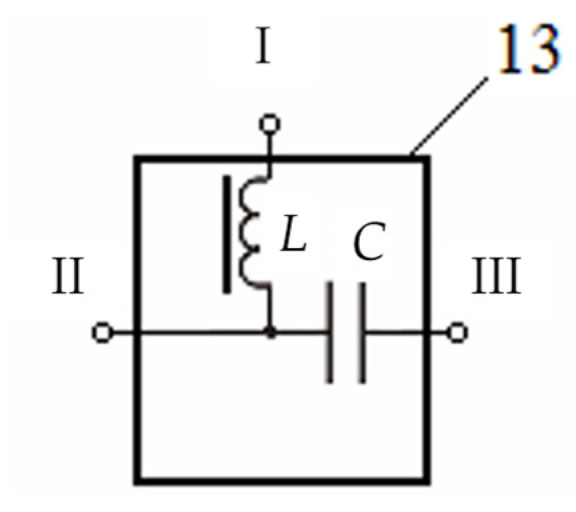
A version of the technical implementation of the separating filtration.

Figure 24 shows a version of the technical implementation of procedure 13, where *L* and *C* are the reactive components of the procedure, ensuring the corresponding separation of the *U*_MMC_ data signal in the MMC (HF filtering using a capacitor *C*) and the *U*_0_ electric bias signal (LF filtering using a choke in the form of inductance *L*), i.e., *U_MMC_* enters the MMC through the HF separation filter, and the *U*_0_ constant scaled electrical signal is fed to the FMA choke through the separation LF filter.

When implementing the multiplied detection technique, the operating frequency signal *U_H_* is fed from audio frequency generator 6 to LA 2, inducing a primary electromagnetic field in the surrounding space. The primary electromagnetic field is generated and the secondary one is recorded through spatial combination of LA 1 and receiving FA 2. Since the LA and FA have a mutual orthogonal arrangement, then in the absence of the SO in the search area, the total EMF at the FA output will be equal to zero, i.e., *U_MMC_* = 0. Thereat, the AMC output signal will be the maximum for a given *U_H_* value: *U_AMC_* = max. Therefore, in the absence of SO 8 in the search area, the signal ratio Δ = *U_MMC_*/*U_AMC_* will be equal to zero: Δ = 0.

When SO 8 occurs in the host medium 7, the LA’s *H_P_* primary field induces an EMF in it, which causes eddy currents 9 generating a secondary (reradiated) electromagnetic field with a polarized magnetic component *H_S_*. Thus, in the presence of PO 8 in the host medium, a horizontal magnetic component *H_X_* of the secondary magnetic field occurs, violating the initial compensation. The presence of SO in the host medium, sharply contrasting against its background with its physical properties, also correspondingly redistributes the existing ratio Δ between the *H_X_* and *H_Y_* components in the *H_S_* secondary magnetic field composition, i.e., in the general case, Δ is a variable taking on a certain numerical value in the presence of SO at its well-pronounced polarizing properties.

In other words, a stable local inhomogeneity Δ in the form of SO in the host medium will take on a certain threshold value ∆*_T_*, which is a factor reliably determining the presence or absence of a foreign SO in the host medium. In this case, a noticeable increase in the *H_X_* component and a corresponding change in *H_Y_*, i.e., a significant increase in Δ to the conditional threshold value ∆*_T_* will be recorded.

The horizontal magnetic component *H*_X_ of the secondary magnetic field *H_S_* acts on receiving FA 2 and induces an induction EMF in it in the form of the MMC data signal *U_MMC_*, which undergoes separating filtration 13 with subsequent recording and in-phase-quadrature conversion 3.

Eddy currents 9, induced in SO 8, generate a secondary electromagnetic field. That electromagnetic field *H_S_* magnetic component strength will be equal to the strength difference of the magnetic components of the exciting and secondary electromagnetic fields. Thus, the vertical magnetic component *H*_Y_ of the secondary magnetic field *H_S_*, acting on LA 1, will increase its impedance while decreasing the electric current in it. In this case, the impedance of LA 1 will depend on the magnitude and the distribution pattern of eddy currents in SO 8, i.e., its specific electrical conductivity σ and depth *h*. In this case, the informative parameter is the amplitude of the excitation current *I_LA_* of LA 1. The change in the radiating LA 1 impedance, caused by the electromagnetic properties of SO 8, is fixed via an Additional Measuring Channel (AMC). To do this, an electrical signal is read as a voltage proportional to the excitation current *I_LA_* of the radiating LA 1 by the AMC measuring current shunt *R_SH_* and further converted. The resulting signal *U_AMC_* is proportional to the vertical magnetic component *H*_Y_ of the secondary magnetic field. This electrical signal *U_AMC_* is used as the AMC output data signal. Thus, when SO 8 occurs in the search area, the *U*_AMC_ value starts reducing; therefore, the signal ratio *U_MMC_*/*U_AMC_* = Δ will increase.

The technique involves a ratiometric conversion of the data signals *U_MMC_* and *U_AMC_*, the result of which is a quotient of the division *U_MMC_*/*U_AMC_* = Δ. The Δ value is regularly compared with the set threshold ∆*_S_*, which is a factor reliably determining the presence of foreign SO 8 in the host medium 7.

To improve the SO identification and depth definition accuracy, when the fact of the possible SO presence in the host medium is established, i.e., the condition ∆ ≥ ∆*_S_* is met, the FA is switched to the multiplied amplification mode.

When searching for an electrically conductive SO 8, preliminary setting a high MMC sensitivity by switching FA 2 to the multiplied amplification mode may cause false responses from minor host medium abnormalities such as geoelectrical inhomogeneities of the upper earth’s crust layers. This will lead to information uncertainty when implementing the technique in general.

When the condition ∆ ≥ ∆*_S_* is met, the process $F_{\Delta \geq \Delta_{s}}\left(t\right)=1$ occurs, initiating the FA multiplied amplification mode and the algorithmic definition of the SO parameters.

When the process $F_{\Delta \geq \Delta_{s}}\left(t\right)=1$ initiates the FA multiplied amplification mode, the source of a constant scaled electrical signal 12 generates a voltage *U*_0_ of the required magnitude. After separating filtration 13 through the LF filter (choke *L*), this voltage is fed to the FA coil. Due to this, the corresponding direct bias current starts flowing through the FA coil and is used to magnetize the FA core. Thereat, the required current amplitude and reliable separation of the FA coil’s variable data signals from the source of a constant scaled electrical signal *U*_0_ are provided.

Moreover, in separating filtration 13 through the HF filter (capacitor *C*), the electric signal *U*_0_ is separated from the MMC’s secondary conversion circuits, and variable data signals are transmitted from the FA coil to these circuits.

Procedures 12 and 13, in fact, facilitate the magnetization of the FA ferromagnetic core by a constant (bias) magnetic field in the presence of SO 8 in the host medium 7, thereby creating the conditions for switching FA 2 to the multiplied amplification mode when this is the case, which significantly increases the AMC’s data signal *U_MMC_* and thereby, improves the accuracy and sensitivity of the hidden electrically conductive SO detection technique.

In this case, core 2 is, in fact, a magnetic conductor with the parameter μ → μ_max._ controlled by an additional constant bias field *H_bias_*. The measuring coil FA is used here as a control winding connected in series from the side of the source of the control reference input signal *U*_0_ with an LF filter made as a choke with inductance *L*, which has a small active resistance for a DC signal but a large reactive resistance for the variable EMF induced in the FA measuring winding by the measured alternating magnetic field $H_{X}$ and being the FA output signal ${\tilde{U}}_{out}$.

The procedure for recording and in-phase-quadrature conversion of the *U_MMC_* data signal by the main MMC involves decomposing this data signal into in-phase $U_{{IP}_{MMC}}$ and quadrature $U_{Q_{MMC}}$ electrical signals while the in-phase-quadrature conversion is synchronized with the time parameters of the harmonic signal *U_H_* of sinusoidal voltage generator 6, exciting the primary electromagnetic field, using corresponding synchronization process *F*(ω; φ; *t*). In this case, the received signal $U_{{IP}_{MMC}}$ is proportional to the specific electrical conductivity σ*_SO_* of SO 8, and the $U_{Q_{MMC}}$ signal is proportional to the magnetic susceptibility μ*_SO_* of SO 8. These in-phase and quadrature electrical signals are used as the MMC output signals.

Under appropriate conditions (*H_bias_* = *H*_0_), the FA operation in the multiplied amplification mode significantly reduces the error of the real conversion of the input *H_X_* value by the main measuring channel and provides the highest sensitivity of the AM.

The mode of the algorithmic definition of the SO parameters initiates procedure 5 for recording and joint algorithmic processing of output data signals $U_{{IP}_{MMC}},$ $U_{Q_{MMC}}$, and *U_MMC_*, the results of which determine the subsurface SO 8 depth *h_SO_* in the search area and identify it by defining its magnetic permeability μ*_SO_* and electrical conductivity σ*_SO_*. All the components required for the data signal processing algorithm are defined at the preliminary preparation stage by exposing the FA to a certain set of reference standards.

Unit 5 determines the SO depth *h_SO_* and finally identifies the SO by the μ*_SO_* and σ*_SO_* values using the following algorithms:

(5) $$\begin{array}{c} \sigma_{SO}=\frac{1}{\left(1\pm \xi_{\sigma }/\mu_{max})\cdot (a_{2}^{*}\cdot b_{1}^{*}-a_{1}^{*}\cdot b_{2}^{*}\right)}\cdot (b_{1}^{*}\cdot U_{{IP}_{MMC}}-b_{2}^{*}\cdot U_{Q_{MMC}});\\ \mu_{SO}=\frac{1}{\left(1\pm \xi_{\mu }/\mu_{max})\cdot (a_{2}^{*}\cdot b_{1}^{*}-a_{1}^{*}\cdot b_{2}^{*}\right)}\cdot (a_{2}^{*}\cdot U_{Q_{MMC}}-a_{1}^{*}\cdot U_{{IP}_{MMC}});\\ h_{SO}=\frac{U_{AMC}}{b_{3}^{*}}-\frac{a_{3}^{*}\cdot (b_{1}^{*}\cdot U_{{IP}_{MMC}}+b_{2}^{1}\cdot U_{Q_{MMC}})}{\left(1\pm \gamma_{\sigma }/\mu_{max})\cdot b_{3}^{*}\cdot (a_{2}^{*}\cdot b_{1}^{*}-a_{1}^{*}\cdot b_{2}^{*}\right)}. \end{array}$$

where $a_{1}^{*}$, $b_{1}^{*}$ and $a_{2}^{*}$, $b_{2}^{*}$ are the rated static quadrature and in-phase conversion function coefficients of the FA MMC; $a_{3}^{*}$ and $b_{3}^{*}$ are the LA AMC real static function conversion coefficients; ξ_σ_ and ξ_μ_ are sensitivity error coefficients for, respectively, the σ*_SO_* and μ*_SO_* parameters.

According to the provided algorithms, when ∆ ≥ ∆*_T_*, the FA is switched to the multiplied amplification mode, where ξ_σ_/μ_max_→0 and ξ_μ/_μ_max_→0, i.e., the FA real static conversion function approaches its rated form.

Introducing a controlled multiplied FA amplification mode noticeably improves the technique’s sensitivity in general, which significantly reduces the likelihood of such consequences as target drop-out or false response, i.e., the lowest possible values of the measured σ*_SO_* and μ*_SO_* provide the stable mode of detecting and identifying SOs.

**Figure 25:**
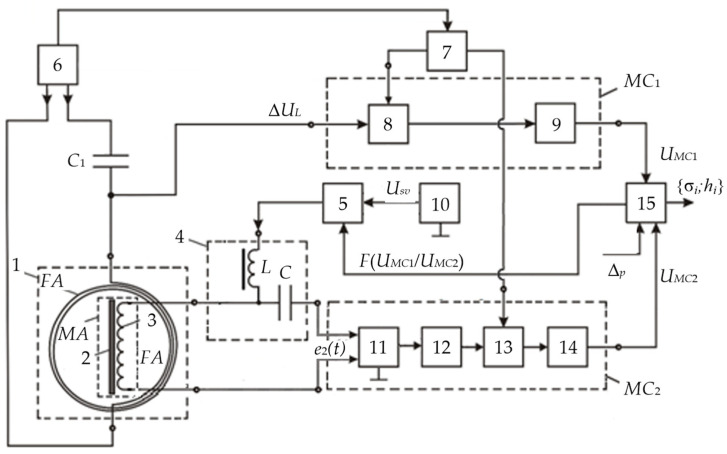
Structure flowchart of a metal detector.

Figure 25 shows a structure flowchart of a metal detector implementing the multiplied subsurface sounding technique to detect metal SOs, where 1—AM; 4—separating filter (SF); 5—switch; 6—audio frequency electrical signal generator; 7—reference voltage generator; MC_1_ and MC_2_—the first and second measuring channels, respectively; 10—magnetic amplification mode setting voltage generator; 15—computing unit (CU).

AM contains a transmitting-and-receiving loop antenna LA and a receiving magnetic antenna FA consisting of a ferrite core 2 and a receiving coil 3. LA has an orthogonal spatial arrangement relative to the receiving coil 3. A capacitor C_1_ is connected in series with LA 2, and they jointly form a resonant circuit. The first MC_1_ contains a synchronous detector 8 and a secondary signal processing unit 9. In turn, MC_2_ includes an amplifier with a symmetrical input 11, a selective amplifier 12, a synchronous detector 13, and a secondary signal processing unit 14.

It should be noted that there is also a modified version of the technical implementation of the multiplexed subsurface sounding technique for detecting metal SOs, where the AM contains a two-section FA with a differential connection of these section windings [47].

**Figure 26:**
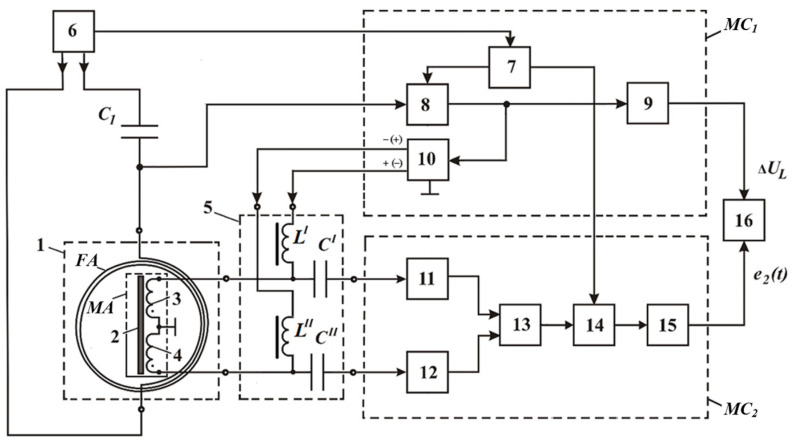
Structure flowchart of a modified metal detector version.

Figure 26 shows a structure flowchart of such an improved metal detector, where 1—AM; 5—separating filter (SF); 6—audio frequency electrical signal generator; 7—reference voltage generator; MC_1_ and MC_2_—the first and second measuring channels, respectively; 10—magnetic amplification mode setting voltage generator; 16—switch.

AM 1 of the modified metal detector contains a transmitting-and-receiving loop antenna LA and a receiving two-section FA consisting of a ferrite core 2 and two identical receiving coils 3 and 4 with differentially connected windings. In the presence of external in-phase electromagnetic noise, inducing EMFs with the same amplitudes and phases in coils 3 and 4, the specified connection of coils 3 and 4 will provide complete mutual compensation for these ENFs. SF 5 consists of two filtration subsystems, one of which contains reactive elements *L*′ and *C*′, and another—reactive elements *L*″ and *C*″.

The first MC1 contains a synchronous detector 8 and a secondary signal processing unit 9, and MC2—buffer amplifiers 11 and 12, an instrumental amplifier 13, a synchronous detector 14, and a secondary signal processing unit 15. Unit 10 generates bipolar mode setting voltages (−*U_MS_* and +*U_MS_*), which are fed to the data inputs of switch 16. When switch 16 is activated by the *U_MS_*(*t*) signal, the mode setting voltages from its data outputs excite corresponding bias currents of the MA coils 3 and 4 through the corresponding reactive elements (*L*′ and *L*″) of SF 5. Thereat, the required phases and amplitudes of these currents and reliable spacing of the unit 16 outputs from variable data signals from coils 3 and 4 are provided. Moreover, the inputs of buffer amplifiers 11 and 12 are separated from constant electrical signals from unit 16 through the corresponding reactive elements (*C*′ and *C*″) of SF 5.

**Figure 27:**
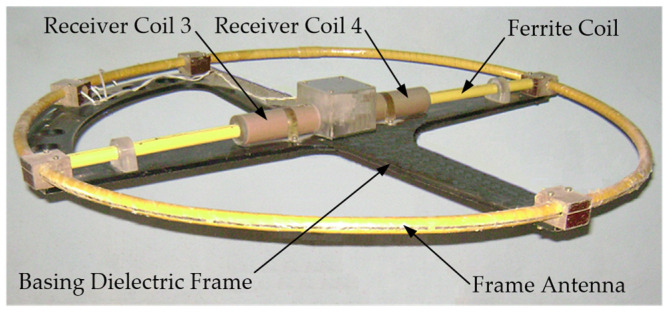
AM layout of a modified metal detector.

Figure 27 shows a general AM layout of a modified version of the metal detector.

The modified metal detector version implementing the multiplied subsurface sounding technique facilitates high efficiency and simplicity of primary field compensation and significantly reduces inter-turn leakage in the FA coils, exposure to external electromagnetic noise, and the impact of changes in the receiving coil and ferromagnetic core parameters on the metal detector output characteristics. Thus, the modified metal detector version has many advantages over its analog.

The multiplied induction sounding technique provides a high level of efficiency in detecting and identifying various subsurface objects. The implementation of a resonant excitation mode in the circuit of the transmitting-and-receiving radiating loop antenna LA, as well as the use of two independent measuring channels and switching the receiving FA to the magnetic amplifier mode, the control signal for which is obtained using the LA, improves the sounding sensitivity and accuracy and leads to a significant decrease in the sensitivity threshold of the FA functioning as a measuring transducer. This allows for recommending this technique for use in the quick detection and precise location of various SOs during construction, earthworks, rescue, repair, etc.

### 6.4. Metal Detector with Goniometric AM

Wide opportunities in choosing the type and spatial orientation of radiating LAs and receiving antennas allow for solving numerous problems arising when detecting, tracking, and examining SOs using electromagnetic impedance measurements. An example is determining on which AM side an extended SO (e.g., an electric cable) passes by measuring secondary electromagnetic fields and finding the angle α, at which the AM crosses the electric cable route in the tracking mode.

**Figure 28:**
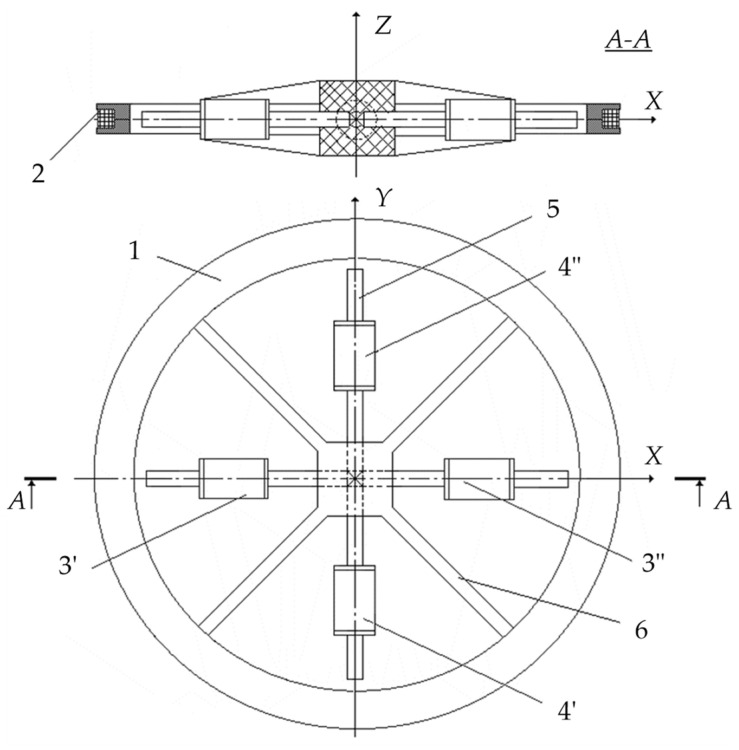
The AM goniometric metal detector design.

The analysis of the design features of Ams with different LA arrangements showed the expediency of using a set of crossed receiving FAs placed inside and in the plane of the horizontal radiating LA (coplanar). An example of such a technical solution is a metal detector with a goniometric AM consisting of two mutually perpendicular FAs (Figure 28). The ferrite elements of each FA, forming a cruciform ferrite core 5, respectively, hosts receiving coil sections 3′ ÷ 3″ and 4′ ÷ 4″, having the same shape, size, and number of winding turns and located in pairs symmetrically relative to the AM geometric axes [48,49].

AM consists of LA 2, placed on a flat ring frame 1, and a Bellini–Tosi ferrite antenna combining four elements: a cruciform ferrite core 5, two pairs of sectional coils 3′ ÷ 3″ and 4′ ÷ 4″, forming, respectively, receiving coils 3 and 4, and basing tetraradiate dielectric prism 6. Inside the ring frame 1, in its plane, a basing tetraradiate dielectric prism 6 is placed, the length and height of the rays of which are equal to, respectively, the frame’s inner diameter and approximately the prism thickness. Prism 6 has two symmetrical, transverse, mutually perpendicular through-holes in the center, in which four ferrite elements of the cruciform ferrite core 5 are fixed end-to-end. In each of the pairs, sectional coils 3′ ÷ 3″ and 4′ ÷ 4″ are placed on the corresponding ferrite elements of the cruciform ferrite core 5 symmetrically about the principal symmetry axes of prism 6. This design solution stipulates for a mutually orthogonal arrangement of the generator coil 1 and receiving coils 3, 4. A similar spatial orientation of the generator LA and the FA’s receiving coils meets the condition of initial equality of their mutual induction coefficient to zero.

The LA radiates a radio wave from the starting space point. After some time, this radio wave reaches the SO location point and induces electric currents in it, generating, in turn, reradiated radio waves propagating in all directions, including toward the receiving FA. The reradiated radio wave reaches the FA location point, generating the corresponding electrical signal in the form of an EMF in it.

This EMF is generated as a result of the joint impact of several factors: scattering on the air–ground interface irregularities and soil inhomogeneities; reflection from a subsurface object located at depth *R*; diffraction and scattering of waves on the object surface irregularities, etc. [50,51,52]. All these processes are implemented in some orthogonal coordinate system (X—horizontal, Y—vertical).

RA radiates a radio wave, in which the electric vector ***E****_rad_* has only the X-component ***E****_Xrad_* (horizontal polarization). In the absence of additional limitations, the electric vector of the reflected (reradiated) radio wave ***E****_ref_* will have, in the general case, a different orientation in space than ***E****_rad_*. In other words, in the chosen coordinate system, the ***E****_ref_* field will have two components ***E****_Xref_* and ***E****_Yref_*.

There is a direct proportionality between the intensities of the reflected and radiated radio waves (the lengths of the ***E_ref_*** and ***E****_rad_* vectors), which predetermines the following properties:

***E***_*Xref*_ ∝ ***E***_*Xrad*_; ***E***_*Yref*_ ∝ ***E***_*Xrad*_ or ***E***_*Xref*_ = ***K***_*XX*_ × ***E***_*Xrad*_; ***E***_*Yref*_ = ***K***_*XY*_ × ***E***_*Xrad*_, (6)

where *K_XX_* and *K_XY_* are the respective proportionality factors.

The sounding signal can be conditionally represented as a radio wave:

(7) $$E_{rad}(t)=A(t)\times cos[2\pi f_{0}t+\varphi (t)],$$

where *A*(*t*) and φ(*t*) are functions slowly varying in time over the HF period *T* = 2π/*f*_0_.

In this case, each of the reflected radio wave components will have a certain phase shift relative to the primary radiated radio wave, and the orthogonal components of the reflected radio wave’s electric vector can be dynamically represented as follows:

(8) $$\begin{array}{c} E_{Xref}(t)=K_{XX}\times A(t)\times cos[2\pi f_{0}t+\varphi (t)+\psi_{XX}];\\ E_{Yref}(t)=K_{XY}\times A(t)\times cos[2\pi f_{0}t+\varphi (t)+\psi_{XY}]. \end{array}$$

When the SO is irradiated with a horizontally polarized radio wave, the reflected radio wave is defined by four parameters characterizing the SO: *K_XX_*, *K_XY_*, ψ*_XX_*, and ψ*_XY_*. For a radiating radio wave with only a Y-component (vertically polarized radio wave), four SO characteristics will be obtained: *K_YY_*, *K_YX_*, ψ*_YY_*, ψ*_YX_.*

Thus, if the emitted radio wave has an arbitrary polarization, i.e., there are two electric vector components ***E****_Xrad_* and ***E****_Yrad_*, then the SO can be completely described using the eight aforementioned parameters.

Considering the presence of the SO polarized basis and mutually orthogonal polarized bases of the LA and FA, the polarization vectors of back reflection scattering from the SO, i.e., the amplitude received signal, are as follows:

(9) $${\dot{S}}_{kl}(t)=\sqrt{\sigma_{kl}}\times e^{i\phi_{kl}(t)},$$

where ${\dot{S}}_{kl}(t)$ is the complex amplitude of the signal received from the underlying surface during irradiation and reception at, respectively, the *l*-th and *k*-th orthogonal polarizations; k,l=(1÷2), σ*_kl_* and φ*_kl_* are the effective scattering surface and the object surface phase during irradiation and reception at, respectively, the *l*-th and *k*-th orthogonal polarizations.

The FA receives reflected radio waves mainly within a certain solid angle ∆Φ (viewing angle) that can be quantitatively evaluated using two plane angles ∆α and ∆β in two mutually perpendicular sections of that solid angle, the values of which are defined by the λ/*d* ratio of the wavelength λ to the antenna’s linear size *d* in the corresponding sections. In this case, currents arise at the FA output, generated by electric currents excited by an incident wave on a rectangular area with linear dimensions R∆α × R∆β, located at a distance R from the FA.

**Figure 29:**
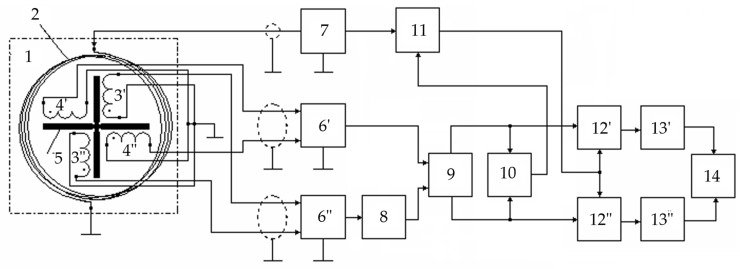
Structure flowchart of a goniometric metal detector.

Figure 29 shows the structure flowchart of a goniometric metal SO detector.

On Figure 29: 1—AM; 6′ and 6″—channel differential amplifiers; 7—audio frequency AC generator; 8—phase shifter; 9—sum-difference device; 10—range phase shifter; 11—controlled reference voltage generator; 12′ and 12″—channel synchronous detectors; 13′ and 13″—channel ADCs; 14—recorder. AM 1 comprises radiating LA 2; goniometric FA consists of two pairs of sectional receiving coils (3′, 3″ and 4′, 4″) and a cruciform ferrite core 5.

Radiating LA 2 has a mutually orthogonal arrangement relative to the receiving coils of the goniometric ferrite antenna, which provides the required geometric compensation for the primary field, and the differential connection of each corresponding pair of sectional receiving coils implements mutual compensation for in-phase EMFs induced in them by external electromagnetic noise.

An operating frequency signal is fed from the audio frequency generator 7 to the generator coil 2, creating a primary alternating magnetic field in the surrounding space. The generator coil field in the form of a horizontally polarized wave from the vertical direction magnetizes the environment (the host medium and the SO), generating an alternating secondary magnetic field in the form of polarization scattering vectors of the back reflection from the SO, which is perceived by the receiving coils of the goniometric ferrite antenna.

Therefore, at any considered time instant, radio waves reflected from various electrically conductive objects located at a distance *R* from the receiving point generate signals at the metal detector output.

Thereat, the measured parameters are the induction components of either the alternating magnetic field or the alternating electric field intensity, or their modules.

In turn, the signals from each FA are fed to the corresponding channel differential amplifiers 6′ and 6″. The output of the differential amplifier 6′ is connected directly to the first input of the sum-difference device 9, and the output of the differential amplifier 6” is connected to the second input of the sum-difference device 9 through the phase shifter 8 ensuring a constant phase shift of 90° over the entire operating range.

At the sum-difference device 9 output, two voltages are generated:

*U_A_* = *U*_1_ + i*U*_2_ and *U_B_* = *U*_1_ − *iU*_2_, (10)

where *U*_1_ = *U × cos*φ and *U*_2_ = *U × sin*φ are voltages from the first and second ferrite antennas, respectively; *U* is the voltage from the first and second FAs when they are oriented to the signal maximum.

Voltages *U_A_* and *U_B_* are fed to the range phase shifter 10 implementing the Directional Pattern (DP) control principle based on the use of a broadband amplifier and phase shifter and thereby ensuring the possibility of changing the phase shift in the operating range within ±90°.

It should be noted that the voltage at the range phase shifter 10 output is defined by the equation:

*U*_P_ = *A × sin*(φ + ψ), (11)

where *A* is a constant depending on the voltages *U*_1_ and *U*_2_ and the amplifier gain.

In this case, the maximum reception is obtained with φ + ψ = ±90°, and there is no reception at φ = −ψ. This facilitates the DP control in the horizontal plane, which is almost similar to the FA rotation.

Moreover, the voltages *U_A_* and *U_B_* from the sum-difference device 9 outputs are fed to the corresponding data inputs of the synchronous channel detectors 12′ and 12″, the reference inputs of which are connected to the controlled reference voltage shaper 11 output.

The voltage from the range phase shifter 10 output is fed to the second (control) input of the reference voltage generator 11. The reference voltage phase for synchronous detectors is formed according to the control signal from the range phase shifter 10 from a sinusoidal voltage fed from the generator 7 output to the first input of unit 11. In this case, the interfering signal coming from different angular directions is significantly reduced without a noticeable suppression of the useful signal, allowing one to obtain sample values of the polarization vectors of the back reflection scattering from the detected SO.

In turn, the signals from the outputs of the channel synchronous detectors 12′ and 12″ are fed to the corresponding inputs of the channel ADCs 13′ and 13″, converting data signals into digital sequences entered, in turn, into recorder 14. Recorder 14 allows for defining the module and phase of the horizontal magnetic component of the reradiated magnetic field. Based on the values of these informative parameters and the results of comparing the emitted and received radio signals, the SO presence in the host medium and its spatial location are determined.

**Figure 30:**
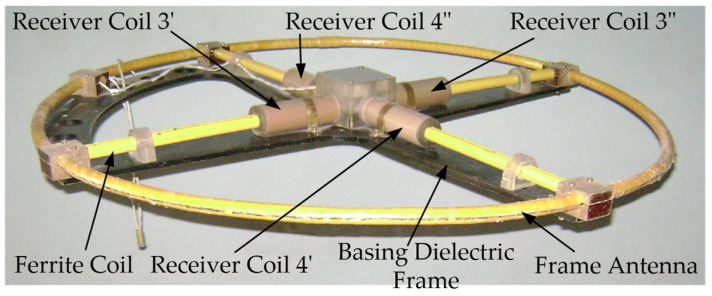
Layout of the AM goniometric metal detector.

Figure 30 shows a general view of the layout of an AM goniometric metal detector.

The Bellini–Tosi antenna allows for isolating interfering radio stations by setting the zero DP direction on it, significantly reducing the direct atmospheric noise. In turn, placing two spaced and sequentially connected sectional inductance coils on the corresponding composite ferrite rod of each FA improves the metal detector efficiency, i.e., increases the active FA height and sensitivity. Moreover, in this case, the FA inductance reduces almost twice, which allows for increasing the total number of turns in both coils with pull-through winding by about $\sqrt{2}$.

Other advantages of this metal detector are the efficiency and simplicity of the applied way of compensation, the eliminated impact of variations in the ferromagnetic core parameters and receiving coils on the metal detector characteristics, and significantly reduced inter-turn leakage in the receiving coils and the impact of external noise on them. A successful combination of the advantages of various well-known AM classes and the FA features favorably distinguishes the considered metal detector from its analogs.

## 7. Conclusions

An overview of scientific literature data on the basic modern equipment for detecting various metal and metal-containing subsurface objects has been performed. Known technical solutions have been comparatively analyzed. 

It is shown that any surface object search system contains two functional modules: 1—antenna module (primary measuring transducer), designed to search for SO by generating a primary alternating electromagnetic field, registering the reradiated electromagnetic field from this SO and converting this field into an electrical output information signal; 2—electronic module (secondary measuring transducer) for recording, processing the information signal and the final information output. At the same time, the capabilities of conventional metal detectors are almost completely exhausted; thus, their improvement ways are based mainly on enhancing the procedures for processing the information signal in the secondary measuring transducer. Therefore, special attention was paid to promising versions of metal detectors built using new physical operation principles of the primary measuring transducer. Their design and operation are analyzed in detail, the advantages and prospects for use are described. It is shown that, for example, a hybrid method of subsurface sounding is implemented by arranging three measuring channels: two measuring channels are made by the receiving FA (to measure the current value of the voltage amplitude of the active component of the input signal and the current value of the voltage amplitude of the reactive component of the input signal), and the third measuring body channel is made by the transceiver RA (it is a channel for measuring the current value of the amplitude of the exciting current RA). Such information redundancy significantly increases the reliability and accuracy of the method for detecting hidden metal objects and increases the efficiency of subsurface sounding in general.

Moreover, worthy of special attention is the multiplicative method of induction sounding implemented by creating a resonant excitation mode in the circuit of the receiving-transmitting frame antenna RA, as well as by using two independent measuring channels and transferring the receiving FA into the magnetic amplifier mode. This provides for improved sounding sensitivity and accuracy and also significantly reduces the FA sensitivity threshold that performs the functions of a measuring transducer. The designs of metal detectors using ferrite antennas, as well as a goniometric antenna module consisting of two mutually perpendicular ferrite antennas, are also very promising. Such metal detectors feature a successful combination of the advantages of various known classes of antenna modules and the peculiarities of ferrite antennas with minimal exposure to external interference. These types of metal detectors are worthy of further research and wide application.

This paper shows that the active and dynamic development of metal detectors is determined by the increasing attractiveness of such systems for solving applied problems in detecting and identifying underground utilities (underground cable lines, pipelines, heating networks) and individual hidden objects (buried scrap metal, treasures, hidden shells, mines, etc.). The high market demand makes appropriate theoretical and experimental research on metal detectors very relevant and increasingly desirable.
